# Supplementary material for: Recombinant zoster vaccine is associated with a reduced risk of dementia
Source: Nat Commun. 2026 Feb 9;17:2056. doi: 10.1038/s41467-026-69289-0 (PMC12949228; doi:10.1038/s41467-026-69289-0)
Supplement: Supplementary file 1 — Supplementary Information [file 41467_2026_69289_MOESM1_ESM.pdf]

Supplementary Materials for:

Recombinant zoster vaccine is associated with a reduced risk of dementia

Emily Rayens, Lina S. Sy, Lei Qian, Bradley K. Ackerson, Julia Tubert, Yi Luo, Punam P. Modha, Raul O. Calderon, Elizabeth Chmielewski-Yee, Driss Oraichi, Huifeng Yun, Carol Koro, Hung Fu Tseng

This file includes:

Supplementary Tables 1 – 19

Supplementary Figures 1 – 5

Supplementary Methods

Supplementary References

Supplementary Materials

Table of Contents

Supplementary Table 1. Chart validation of dementia and MCI diagnoses.

Supplementary Table 2. Baseline characteristics of the two-dose RZV-vaccinated and unvaccinated cohorts before weighting.

Supplementary Figure 1. Comparison of absolute standardized difference before and after IPTW among two-dose RZV-vaccinated versus unvaccinated individuals.

Supplementary Table 3. Incidence rate and hazard ratio of dementia among two-dose RZV-vaccinated versus unvaccinated individuals using Cox regression model with IPTW.

Supplementary Table 4. Incidence rates of dementia by HZ status.

Supplementary Table 5. Baseline cohort characteristics of the two-dose RZV-vaccinated and Tdap-vaccinated cohorts before weighting.

Supplementary Figure 2. Comparison of absolute standardized difference before and after IPTW among two-dose RZV-vaccinated versus Tdap-vaccinated individuals.

Supplementary Table 6. Baseline cohort characteristics of the two-dose RZV-vaccinated and Tdap-vaccinated cohorts after weighting.

Supplementary Table 7. Incidence rate and hazard ratio of dementia among RZV-vaccinated versus unvaccinated or Tdap-vaccinated individuals using Cox regression model with IPTW.

Supplementary Table 8. Incidence rate and hazard ratio of NCO in two-dose RZV-vaccinated versus unvaccinated individuals using Cox regression model with IPTW.

Supplementary Table 9. Baseline characteristics of two-dose RZV-vaccinated and unvaccinated cohorts for MCI analysis before weighting.

Supplementary Table 10. Baseline characteristics of two-dose RZV-vaccinated and unvaccinated cohorts for MCI analysis after weighting.

Supplementary Table 11. Incidence rate and hazard ratio of MCI in two-dose RZV-vaccinated versus unvaccinated individuals using Cox regression model with IPTW.

Supplementary Figure 3. Cumulative incidence of MCI among two-dose RZV-vaccinated versus unvaccinated individuals.

Supplementary Table 12. Time to diagnosis of dementia from MCI diagnosis.

Supplementary Table 13. Baseline characteristics of two-dose RZV-vaccinated and unvaccinated cohort with index date prior to COVID-19 pandemic after weighting.

Supplementary Table 14. Incidence rate and hazard ratio of outcomes for sensitivity analyses among two-dose RZV-vaccinated versus unvaccinated individuals using Cox regression model with IPTW.

Supplementary Table 15. Baseline characteristics of two-dose RZV-vaccinated and unvaccinated cohort without censoring receipt of HZ vaccines during follow-up after weighting.

Supplementary Table 16. Incidence rate and hazard ratio of dementia with follow-up starting 1 year after index date among two-dose RZV-vaccinated versus unvaccinated or Tdap-vaccinated individuals using Cox regression model with IPTW.
Supplementary Figure 4. Cumulative incidence estimates of dementia among two-dose RZV-vaccinated versus unvaccinated individuals with follow-up starting at 1 year after index date.
Supplementary Figure 5. Cumulative incidence estimates of dementia among two-dose RZV-vaccinated versus Tdap-vaccinated individuals with follow-up starting 1 year after index date.
Supplementary Methods
Supplementary Table 17. Outcome definition ICD-10 codes.
Supplementary Table 18. Medications for the management of dementia symptoms.
Supplementary Table 19. Covariate ICD-10 codes.
Supplementary References

Supplementary Table 1. Chart validation of dementia and MCI diagnoses.

Outcomes	Reviewed Cases	Confirmed Cases[a]	PPV (95% CI)
Dementia (≥1 dementia diagnosis)	100	94	94.0% (87.4%–97.8%)
Alternative dementia definition			
≥2 dementia diagnoses	74	71	95.9% (88.6%–99.2%)
≥1 dementia diagnosis with dementia medication	33	32	97.0% (84.2%–99.9%)
≥2 dementia diagnoses or ≥1 dementia diagnosis with dementia medication	76	73	96.1% (88.9%–99.2%)
MCI (≥1 MCI diagnosis)	100	98	98.0% (93.0%–99.8%)

[a]Charts were reviewed to determine if the individual was diagnosed with dementia or MCI by the provider on the date the diagnosis code was applied. Cases in which the individual was diagnosed with dementia or MCI, but the diagnosis was not confirmed by a specialist (neurologist, gerontologist, psychiatrist, or psychologist) to whom individuals were referred for further evaluation were considered to be not confirmed.

CI, confidence interval; MCI, mild cognitive impairment; PPV, positive predictive value

Supplementary Table 2. Baseline characteristics of the two-dose RZV-vaccinated and unvaccinated cohorts before weighting.

	Vaccinated n=65,800	Unvaccinated n=263,200	ASD
Age at index date, years			0.020
Mean (SD)	73.23 (6.15)	73.36 (6.70)	
Median	72	72	
Q1, Q3	68, 77	68, 77	
Minimum, Maximum	65, 101	65, 109	
Age at index date, years, n (%)			0.000
65–69	21,229 (32.3)	84,916 (32.3)	
70–74	20,753 (31.5)	83,012 (31.5)	
75–79	13,161 (20.0)	52,644 (20.0)	
≥80	10,657 (16.2)	42,628 (16.2)	
Sex, n (%)			0.000
Female	37,948 (57.7)	151,792 (57.7)	
Male	27,852 (42.3)	111,408 (42.3)	
Race/Ethnicity, n (%)			0.000
Non-Hispanic White	40,309 (61.3)	161,236 (61.3)	
Non-Hispanic Black	2994 (4.6)	11,976 (4.6)	
Hispanic	10,380 (15.8)	41,520 (15.8)	
Non-Hispanic Asian	10,522 (16.0)	42,088 (16.0)	
Other/Multiple/Unknown	1595 (2.4)	6380 (2.4)	
History of ZVL vaccination[a], n (%)			0.000
No	22,120 (33.6)	88,480 (33.6)	
Yes, ≤5 years	5716 (8.7)	22,864 (8.7)	
Yes, >5 years	37,964 (57.7)	151,856 (57.7)	
MCI status[b], n (%)			0.023
No	64,744 (98.4)	258,199 (98.1)	
Yes	1056 (1.6)	5001 (1.9)	
History of HZ[a], n (%)			0.143
No	56,029 (85.2)	230,914 (87.7)	
Yes, ≤2 years	3448 (5.2)	6545 (2.5)	
Yes, >2 years	6323 (9.6)	25,741 (9.8)	
Length of continuous membership[a], years, n (%)			0.101
1–<5	8048 (12.2)	40,050 (15.2)	
5–<10	10,983 (16.7)	47,411 (18.0)	
≥10	46,769 (71.1)	175,739 (66.8)	
Other vaccines[c], n (%)			
Influenza vaccine	60,045 (91.3)	209,722 (79.7)	0.333
COVID-19 vaccine	1 (0.0)	21 (0.0)	0.009
Other[d]	21233 (32.3)	57,627 (21.9)	0.235
Number of outpatient and virtual visits[c], n (%)			0.307
0–4	7553 (11.5)	60,185 (22.9)	
5–10	22,393 (34.0)	81,716 (31.0)	
≥11	35,854 (54.5)	121,299 (46.1)	

Number of Emergency Department visits[c], n (%)			0.098
0	54,510 (82.8)	209,094 (79.4)	
1	8133 (12.4)	36,336 (13.8)	
≥2	3157 (4.8)	17,770 (6.8)	
Number of hospitalizations[c], n (%)			0.099
0	62,327 (94.7)	243,548 (92.5)	
1	2812 (4.3)	14,530 (5.5)	
≥2	661 (1.0)	5122 (1.9)	
Preventive care[c], n (%)	42,230 (64.2)	140,043 (53.2)	0.224
Comorbidities[c], n (%)			
Kidney disease	10,672 (16.2)	46,317 (17.6)	0.037
Heart disease	4653 (7.1)	23,937 (9.1)	0.074
Lung disease	11,328 (17.2)	46,246 (17.6)	0.009
Liver disease	2813 (4.3)	11,174 (4.2)	0.002
Diabetes	15,755 (23.9)	71,851 (27.3)	0.077
Brain tumor	35 (0.1)	256 (0.1)	0.016
Traumatic brain injury	182 (0.3)	1165 (0.4)	0.028
Hearing loss	5122 (7.8)	17,295 (6.6)	0.047
Parkinson's disease	422 (0.6)	2168 (0.8)	0.021
Huntington's disease	4 (0.0)	13 (0.0)	0.002
Multiple sclerosis	125 (0.2)	495 (0.2)	0.000
Anxiety	6996 (10.6)	28,217 (10.7)	0.003
Depression	8421 (12.8)	35,789 (13.6)	0.024
Sleep disorders	9544 (14.5)	34,386 (13.1)	0.042
Metabolic syndrome	5540 (8.4)	25,917 (9.8)	0.050
Hypertension	38,170 (58.0)	149,471 (56.8)	0.025
Alcohol abuse	865 (1.3)	4124 (1.6)	0.021
Dyslipidemia	48,284 (73.4)	171,201 (65.0)	0.181
Viral infections	2861 (4.3)	10,028 (3.8)	0.027
Immunocompromised at index date[e], n (%)	3436 (5.2)	12,474 (4.7)	0.022
Charlson comorbidity score[c,f]			0.057
Mean (SD)	1.66 (1.93)	1.78 (2.10)	
Median	1	1	
Q1, Q3	0, 2	0, 3	
Minimum, Maximum	0, 17	0, 17	
Charlson comorbidity score[c,f], n (%)			0.081
0	22,908 (34.8)	96,332 (36.6)	
1	15,806 (24.0)	54,429 (20.7)	
≥2	27,086 (41.2)	112,439 (42.7)	
Frailty index[c,g]			0.095
Mean (SD)	0.13 (0.03)	0.14 (0.04)	
Median	0.13	0.13	
Q1, Q3	0.11, 0.15	0.11, 0.16	
Minimum, Maximum	0.04, 0.37	0.05, 0.40	

Frailty index[c,g], n (%)			0.095
Q1	16,009 (24.3)	66,232 (25.2)	
Q2	17,947 (27.3)	64,303 (24.4)	
Q3	17,178 (26.1)	65,075 (24.7)	
Q4, most frail	14,666 (22.3)	67,590 (25.7)	
Smoking[c], n (%)			0.305
No	48,072 (73.1)	171,552 (65.2)	
Yes	16,100 (24.5)	66,758 (25.4)	
Unknown	1628 (2.5)	24,890 (9.5)	
Body mass index[h], n (%)			0.315
<18.5	894 (1.4)	3887 (1.5)	
18.5–<25	21,491 (32.7)	71,357 (27.1)	
25–<30	24,270 (36.9)	87,582 (33.3)	
≥30	17,368 (26.4)	74,441 (28.3)	
Unknown	1777 (2.7)	25,933 (9.9)	
Neighborhood median household income, n (%)			0.189
<$40,000	2046 (3.1)	12,189 (4.6)	
$40,000–$59,999	8516 (12.9)	45,276 (17.2)	
$60,000–$79,999	13,183 (20.0)	59,400 (22.6)	
≥$80,000	41,997 (63.8)	145,566 (55.3)	
Unknown	58 (0.1)	769 (0.3)	
Neighborhood-level education[i], n (%)			0.168
≤High school	9519 (14.5)	54,029 (20.5)	
>High school	56,225 (85.4)	208,412 (79.2)	
Unknown	56 (0.1)	759 (0.3)	
Medicaid, n (%)	1796 (2.7)	10,236 (3.9)	0.065
Year of index date, n (%)			0.000
2018	8153 (12.4)	32,612 (12.4)	
2019	34,932 (53.1)	139,728 (53.1)	
2020	22,715 (34.5)	90,860 (34.5)	
Years of follow-up[j]			N/A
Mean (SD)	3.40 (0.99)	1.81 (1.27)	
Median	3.60	1.90	
Q1, Q3	2.88, 4.02	0.52, 2.78	
Minimum, Maximum	0.00, 5.22	0.00, 5.02	
Individuals appear in both exposure groups, n (%)	25,987 (39.5)	25,987 (9.9)	N/A

[a]Defined based on all available medical records prior to the index date.
[b]Defined based on all available medical records prior to index date to 6 months after the index date.
[c]Defined in the year prior to the index date.
[d]Among subjects who received other vaccines in the year prior to the index date: pneumococcal (60.2%), Tdap/Td (31.8%), hepatitis A or B (10.1%), and other vaccine (13.0%).
[e]Immunocompromised defined as HIV/AIDS, hematopoietic stem cell/solid organ transplant, leukemia/lymphoma, congenital and other immunodeficiencies, or asplenia/hyposplenia at any time prior to the index date, or immunosuppressive medication at index date.
[f]Possible range: 0–29.[1]
[g]Possible range: 0–1.[2]
[h]Defined as most recent in the year prior to the index date.
[i]Defined as <50% or ≥50% of the neighborhood that attained more than high school education

[j]Follow-up started from 6 months after the index date until the end of the follow-up period (31 December 2023), death, receipt of a dose of zoster vaccine, termination of KPSC membership (allowing for a 31-day gap in membership), or outcome of interest, whichever came first. Follow-up for dementia (primary outcome) shown above.

AIDS, acquired immunodeficiency syndrome; ASD, absolute standardized difference; HIV, human immunodeficiency virus; HZ, herpes zoster; KPSC, Kaiser Permanente Southern California; MCI, mild cognitive impairment; n, number; N/A, not applicable; Q, quartile; RZV, recombinant zoster vaccine; SD, standard deviation; Td, tetanus and diphtheria vaccine; Tdap, tetanus, diphtheria, and acellular pertussis vaccine; ZVL, zoster vaccine live

Supplementary Figure 1. Comparison of absolute standardized difference before and after IPTW among two-dose RZV-vaccinated versus unvaccinated individuals.

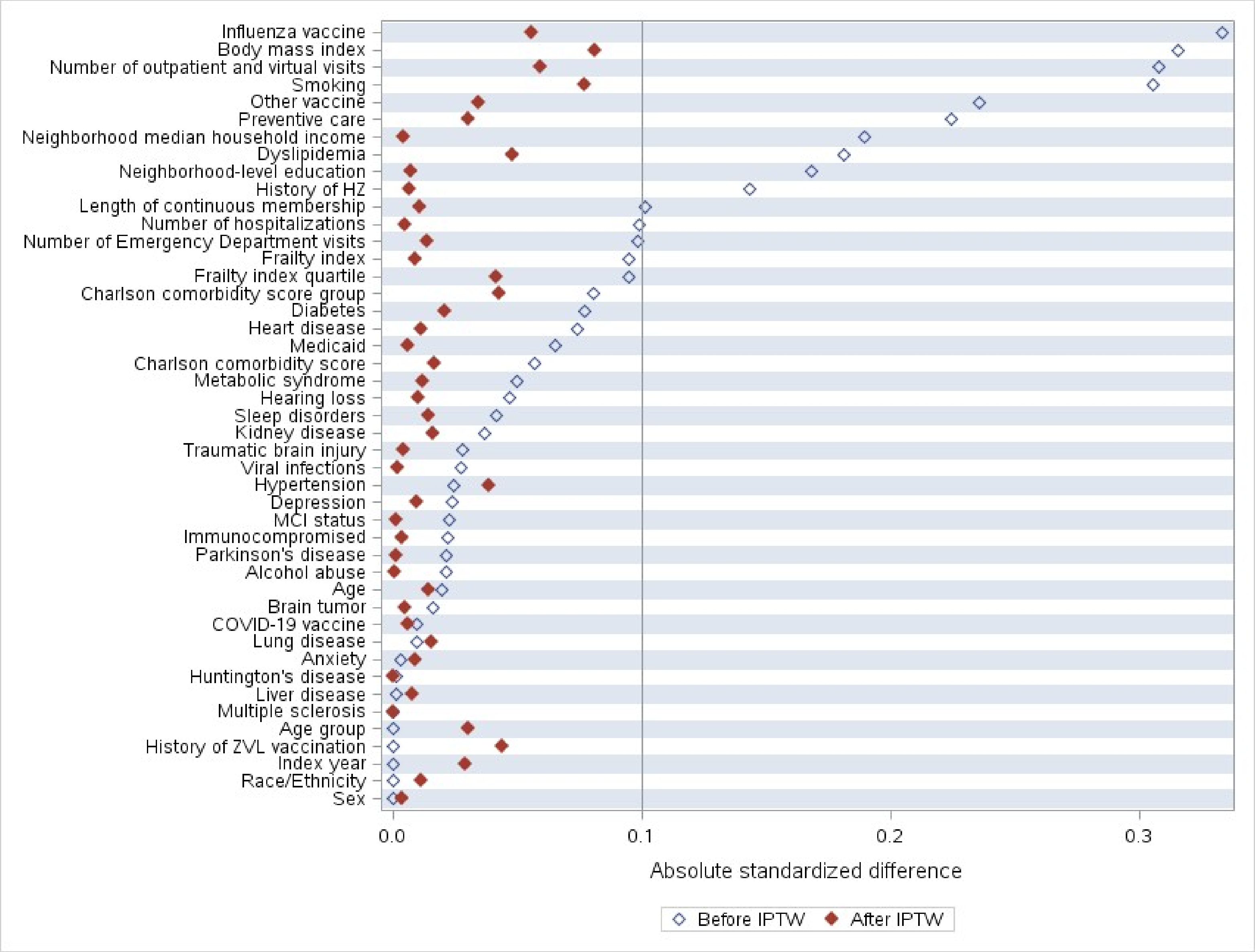

HZ, herpes zoster; IPTW, inverse probability of treatment weighting; MCI, mild cognitive impairment; RZV, recombinant zoster vaccine; ZVL, zoster vaccine live

Supplementary Table 3. Incidence rate and hazard ratio of dementia among two-dose RZV-vaccinated versus unvaccinated individuals using Cox regression model with IPTW.

	RZV-vaccinated				Unvaccinated				Hazard Ratio (95% CI)	
**Dementia**	**n**	**Number of cases**	**Number of person-years**	**Incidence per 1000 person-years (95% CI)**	**n**	**Number of cases**	**Number of person-years**	**Incidence per 1000 person-years (95% CI)**	**Unadjusted**	**Adjusted[a]**
Overall	65,800	2401	223,576.46	10.74 (10.32–11.18)	263,200	10,983	476,623.82	23.04 (22.61–23.48)	0.45 (0.43–0.47)	0.49 (0.46–0.51)
Dementia subtype										
Alzheimer's disease	65,800	717	225,863.06	3.17 (2.95–3.42)	263,200	2796	487,406.86	5.74 (5.53–5.95)	0.49 (0.45–0.53)	0.48 (0.44–0.53)
Vascular dementia	65,800	235	226,387.00	1.04 (0.91–1.18)	263,200	1142	489,461.03	2.33 (2.20–2.47)	0.41 (0.36–0.48)	0.45 (0.39–0.53)
Age at index date, years										
65–69[b]	21,229	187	72,117.43	2.59 (2.25–2.99)	84,916	698	158,036.93	4.42 (4.10–4.76)	0.57 (0.48–0.67)	0.53 (0.44–0.64)
≥70	44,571	2214	151,459.03	14.62 (14.02–15.24)	178,284	10,285	318,586.89	32.28 (31.66–32.92)	0.44 (0.42–0.46)	0.46 (0.44–0.49)
70–79	33,914	1037	117,179.00	8.85 (8.33–9.40)	135,656	4448	248,559.77	17.90 (17.38–18.43)	0.47 (0.44–0.50)	0.49 (0.45–0.53)
≥80[c]	10,657	1177	34,280.02	34.33 (32.44–36.34)	42,628	5837	70,027.12	83.35 (81.21–85.56)	0.40 (0.37–0.42)	0.48 (0.45–0.52)
Sex										
Female	37,948	1311	130,231.01	10.07 (9.54–10.62)	151,792	6579	276,215.17	23.82 (23.25–24.40)	0.41 (0.38–0.43)	0.45 (0.42–0.48)
Male	27,852	1090	93,345.45	11.68 (11.01–12.39)	111,408	4404	200,408.65	21.98 (21.33–22.64)	0.52 (0.49–0.56)	0.55 (0.51–0.59)
Race/Ethnicity, n (%)										
Non-Hispanic White	40,309	1524	137,593.18	11.08 (10.54–11.64)	161,236	7147	284,744.5	25.10 (24.52–25.69)	0.43 (0.40–0.45)	0.46 (0.43–0.48)
Non-Hispanic Black[d]	2994	144	9877.52	14.58 (12.38–17.16)	11,976	694	23,965.16	28.96 (26.87–31.21)	0.50 (0.42–0.60)	0.49 (0.40–0.60)
Hispanic[e]	10,380	364	34,576.80	10.53 (9.50–11.66)	41,520	1668	80,838.31	20.63 (19.66–21.65)	0.50 (0.45–0.56)	0.55 (0.48–0.63)
Non-Hispanic Asian[f]	10,522	327	36,166.49	9.04 (8.11–10.07)	42,088	1333	74,154.69	17.98 (17.03–18.97)	0.48 (0.43–0.55)	0.51 (0.44–0.58)
ZVL status										

Dementia	RZV-vaccinated n	RZV-vaccinated Number of cases	RZV-vaccinated Number of person-years	RZV-vaccinated Incidence per 1000 person-years (95% CI)	Unvaccinated n	Unvaccinated Number of cases	Unvaccinated Number of person-years	Unvaccinated Incidence per 1000 person-years (95% CI)	Hazard Ratio (95% CI) Unadjusted	Hazard Ratio (95% CI) Adjusted[a]
No[g]	22,120	720	72,405.22	9.94 (9.25–10.70)	88,480	3710	187,387.63	19.80 (19.17–20.45)	0.50 (0.46–0.54)	0.51 (0.47–0.56)
Yes	43,680	1681	151,171.24	11.12 (10.60–11.66)	174,720	7273	289,236.19	25.15 (24.57–25.73)	0.42 (0.39–0.44)	0.46 (0.43–0.49)
MCI status										
No	64,744	2103	220,566.27	9.53 (9.14–9.95)	258,199	9470	469,742.19	20.16 (19.76–20.57)	0.45 (0.43–0.48)	0.48 (0.46–0.51)
Yes[h]	1056	298	3010.19	99.00 (88.39–110.87)	5001	1513	6881.63	219.86 (208.31–232.06)	0.50 (0.44–0.56)	0.53 (0.47–0.61)

[a]Adjusted for time-varying vaccination status (influenza, COVID-19, and other vaccines) besides inverse probability of treatment weighting (IPTW).
[b]Adjusted for age in addition to time-varying vaccination status and IPTW.
[c]Adjusted for age and BMI in addition to time-varying vaccination status and IPTW.
[d]More than 20% of covariates were imbalanced (ASD>0.1) within the subgroup. Recalculated propensity scores within the subgroup for IPTW. Baseline COVID-19 vaccination status and Huntington's disease were not included in the propensity score model, due to convergence issues.
[e]Adjusted for BMI, smoking, frailty index, dyslipidemia, number of outpatient and virtual visits, and Charlson comorbidity score in addition to time-varying vaccination status and IPTW.
[f]Adjusted for BMI in addition to time-varying vaccination status and IPTW.
[g]Adjusted for BMI, smoking, number of outpatient and virtual visits, and dyslipidemia in addition to time-varying vaccination status and IPTW.
[h]Adjusted for age, BMI, smoking, number of outpatient and virtual visits, liver disease, and anxiety in addition to time-varying vaccination status and IPTW.

ASD, absolute standard difference; BMI, body mass index; CI, confidence interval; IPTW, inverse probability of treatment weighting; MCI, mild cognitive impairment; n, number; RZV, recombinant zoster vaccine; ZVL, zoster vaccine live

Supplementary Table 4. Incidence rates of dementia by HZ status.

HZ status	RZV-vaccinated				Unvaccinated			
	n	Number of cases	Number of person-years	Incidence per 1000 person-years (95% CI)	n	Number of cases	Number of person-years	Incidence per 1000 person-years (95% CI)
Never had HZ	55,934	1907	189,474.17	10.06 (9.62–10.53)	229,553	9183	412,048.48	22.29 (21.83–22.75)
Had HZ[a,b]	10,348	494	34,102.29	14.49 (13.27–15.82)	37,525	1800	64,575.34	27.87 (26.61–29.20)

[a]Patients with one episode of HZ and those with recurrent HZ were combined for post-hoc analysis due to the small population in the recurrent HZ cohort.
[b]Includes those with one or more episodes of HZ before start of follow-up and/or those who had a first episode during follow-up.

CI, confidence interval; HZ, herpes zoster; n, number; RZV, recombinant zoster vaccine

Supplementary Table 5. Baseline cohort characteristics of the two-dose RZV-vaccinated and Tdap-vaccinated cohorts before weighting.

	RZV-vaccinated n=65,800	Tdap-vaccinated n=65,800	ASD
Age at index date, years			0.033
Mean (SD)	73.23 (6.15)	73.44 (6.32)	
Median	72	72	
Q1, Q3	68, 77	69, 77	
Minimum, Maximum	65, 101	65, 105	
Age at index date, years, n (%)			0.000
65–69	21,229 (32.3)	21,229 (32.3)	
70–74	20,753 (31.5)	20,753 (31.5)	
75–79	13,161 (20.0)	13,161 (20.0)	
≥80	10,657 (16.2)	10,657 (16.2)	
Sex, n (%)			0.088
Female	37,948 (57.7)	35,075 (53.3)	
Male	27,852 (42.3)	30,725 (46.7)	
Race/Ethnicity, n (%)			0.468
Non-Hispanic White	40,309 (61.3)	30,003 (45.6)	
Non-Hispanic Black	2994 (4.6)	6292 (9.6)	
Hispanic	10,380 (15.8)	20,707 (31.5)	
Non-Hispanic Asian	10,522 (16.0)	6994 (10.6)	
Other/Multiple/Unknown	1595 (2.4)	1804 (2.7)	
History of ZVL vaccination[a], n (%)			0.722
No	22,120 (33.6)	41,798 (63.5)	
Yes, ≤5 years	5716 (8.7)	7840 (11.9)	
Yes, >5 years	37,964 (57.7)	16,162 (24.6)	
MCI status[b], n (%)			0.011
No	64,744 (98.4)	64,651 (98.3)	
Yes	1056 (1.6)	1149 (1.7)	
History of HZ[a], n (%)			0.132
No	56,029 (85.2)	57,951 (88.1)	
Yes, ≤2 years	3448 (5.2)	1778 (2.7)	
Yes, >2 years	6323 (9.6)	6071 (9.2)	
Length of continuous membership[a], years, n (%)			0.380
1–<5	8048 (12.2)	17,358 (26.4)	
5–<10	10,983 (16.7)	11,529 (17.5)	
≥10	46,769 (71.1)	36,913 (56.1)	
Other vaccines[c], n (%)			
Influenza vaccine	60,045 (91.3)	51,121 (77.7)	0.381
COVID-19 vaccine	1 (0.0)	3 (0.0)	0.006
Other[d]	21,233 (32.3)	10,682 (16.2)	0.381
Number of outpatient and virtual visits[c], n (%)			0.334
0–4	7553 (11.5)	15,823 (24.0)	
5–10	22,393 (34.0)	19,817 (30.1)	

	RZV-vaccinated n=65,800	Tdap-vaccinated n=65,800	ASD
≥11	35,854 (54.5)	30,160 (45.8)	
Number of Emergency Department visits[c], n (%)			0.156
0	54,510 (82.8)	50,764 (77.1)	
1	8133 (12.4)	9836 (14.9)	
≥2	3157 (4.8)	5200 (7.9)	
Number of hospitalizations[c], n (%)			0.114
0	62,327 (94.7)	60,564 (92.0)	
1	2812 (4.3)	3912 (5.9)	
≥2	661 (1.0)	1324 (2.0)	
Preventive care[c], n (%)	42,230 (64.2)	31,035 (47.2)	0.348
Comorbidities[c], n (%)			
Kidney disease	10,672 (16.2)	11,488 (17.5)	0.033
Heart disease	4653 (7.1)	6243 (9.5)	0.088
Lung disease	11,328 (17.2)	11,128 (16.9)	0.008
Liver disease	2813 (4.3)	2792 (4.2)	0.002
Diabetes	15,755 (23.9)	20,973 (31.9)	0.178
Brain tumor	35 (0.1)	41 (0.1)	0.004
Traumatic brain injury	182 (0.3)	306 (0.5)	0.031
Hearing loss	5122 (7.8)	3929 (6.0)	0.072
Parkinson’s disease	422 (0.6)	543 (0.8)	0.022
Huntington's disease	4 (0.0)	3 (0.0)	0.002
Multiple sclerosis	125 (0.2)	108 (0.2)	0.006
Anxiety	6996 (10.6)	6607 (10.0)	0.019
Depression	8421 (12.8)	8217 (12.5)	0.009
Sleep disorders	9544 (14.5)	7903 (12.0)	0.074
Metabolic syndrome	5540 (8.4)	7777 (11.8)	0.113
Hypertension	38,170 (58.0)	37,772 (57.4)	0.012
Alcohol abuse	865 (1.3)	1005 (1.5)	0.018
Dyslipidemia	48,284 (73.4)	40,161 (61.0)	0.265
Viral infections	2861 (4.3)	2744 (4.2)	0.009
Immunocompromised at index date[e], n (%)	3436 (5.2)	3360 (5.1)	0.005
Charlson comorbidity score[c,f]			0.070
Mean (SD)	1.66 (1.93)	1.80 (2.09)	
Median	1	1	
Q1, Q3	0, 2	0, 3	
Minimum, Maximum	0, 17	0, 17	
Charlson comorbidity score[c,f], n (%)			0.084
0	22,908 (34.8)	23,580 (35.8)	
1	15,806 (24.0)	13,545 (20.6)	
≥2	27,086 (41.2)	28,675 (43.6)	
Frailty index[c,g]			0.102
Mean (SD)	0.13 (0.03)	0.14 (0.04)	
Median	0.13	0.13	

	RZV-vaccinated<br>n=65,800	Tdap-vaccinated<br>n=65,800	ASD
Q1, Q3	0.11, 0.15	0.11, 0.16	
Minimum, Maximum	0.04, 0.37	0.05, 0.37	
Frailty index[c,g], n (%)			0.089
Q1	16,327 (24.8)	16,567 (25.2)	
Q2	17,280 (26.3)	15,625 (23.7)	
Q3	16,876 (25.6)	16,031 (24.4)	
Q4, most frail	15,317 (23.3)	17,577 (26.7)	
Smoking[c], n (%)			0.364
No	48,072 (73.1)	42,101 (64.0)	
Yes	16,100 (24.5)	16,147 (24.5)	
Unknown	1628 (2.5)	7552 (11.5)	
Body mass index[h], n (%)			0.428
<18.5	894 (1.4)	754 (1.1)	
18.5–<25	21,491 (32.7)	14,872 (22.6)	
25–<30	24,270 (36.9)	21,656 (32.9)	
≥30	17,368 (26.4)	20,268 (30.8)	
Unknown	1777 (2.7)	8250 (12.5)	
Neighborhood median household income, n (%)			0.384
<$40,000	2046 (3.1)	4625 (7.0)	
$40,000–$59,999	8516 (12.9)	14,415 (21.9)	
$60,000–$79,999	13,183 (20.0)	16,223 (24.7)	
≥$80,000	41,997 (63.8)	30,459 (46.3)	
Unknown	58 (0.1)	78 (0.1)	
Neighborhood-level education[i], n (%)			0.392
≤High school	9519 (14.5)	20,084 (30.5)	
>High school	56,225 (85.4)	45,641 (69.4)	
Unknown	56 (0.1)	75 (0.1)	
Medicaid, n (%)	1796 (2.7)	4567 (6.9)	0.197
Year of index date, n (%)			0.429
2018	8153 (12.4)	17,715 (26.9)	
2019	34,932 (53.1)	23,607 (35.9)	
2020	22,715 (34.5)	24,478 (37.2)	
Years of follow-up[j]			N/A
Mean (SD)	3.40 (0.99)	2.38 (1.27)	
Median	3.60	2.37	
Q1, Q3	2.88, 4.02	1.38, 3.41	
Minimum, Maximum	0.00, 5.22	0.00, 5.25	
Individuals appear in both exposure groups, n (%)	2118 (3.2)	2118 (3.2)	N/A
Receipt of Tdap among RZV vaccinees, n (%)			N/A
No	889 (1.4)	N/A	
Yes[k]	64,911 (98.6)	N/A	
≥3 years before index date	14,757 (22.7)	N/A	
<3 years prior to or on index date	15,146 (23.3)	N/A	
<3 years after index date	28,396 (43.7)	N/A	

	RZV-vaccinated n=65,800	Tdap-vaccinated n=65,800	ASD
≥3 years after index date	6612 (10.2)	N/A	

[a]Defined based on all available medical records prior to the index date.
[b]Defined based on all available medical records prior to the index date to 6 months after the index date.
[c]Defined in the year prior to the index date.
[d]Among subjects who received other vaccines in the year prior to the index date: pneumococcal (64.7%), Tdap/Td (23.4%), hepatitis A or B (12.2%), and other vaccines (13.9%)
[e]Immunocompromised defined as HIV/AIDS, hematopoietic stem cell/solid organ transplant, leukemia/lymphoma, congenital and other immunodeficiencies, or asplenia/hyposplenia at any time prior to the index date, or immunosuppressive medication at index date.
[f]Possible range: 0–29.[1]
[g]Possible range: 0–1.[2]
[h]Defined as most recent in the year prior to the index date.
[i]Defined as <50% or ≥50% of the neighborhood that attained more than high school education
[j]Follow-up started from 6 months after the index date until the end of the follow-up period (31 December 2023), death, receipt of a dose of zoster vaccine, termination of KPSC membership (allowing for a 31-day gap in membership), or outcome of interest, whichever came first. Follow-up for dementia (primary outcome) shown above.
[k]Most proximal Tdap vaccination through end of follow-up kept to assess timing from index date.

AIDS, acquired immunodeficiency syndrome; ASD, absolute standardized difference; HIV, human immunodeficiency virus; HZ, herpes zoster; KPSC, Kaiser Permanente Southern California; MCI, mild cognitive impairment; n, number; N/A, not applicable; Q, quartile; RZV, recombinant zoster vaccine; SD, standard deviation; Td, tetanus and diphtheria vaccine; Tdap, tetanus, diphtheria, and acellular pertussis vaccine; ZVL, zoster vaccine live

Supplementary Figure 2. Comparison of absolute standardized difference before and after IPTW among two-dose RZV-vaccinated versus Tdap-vaccinated individuals.

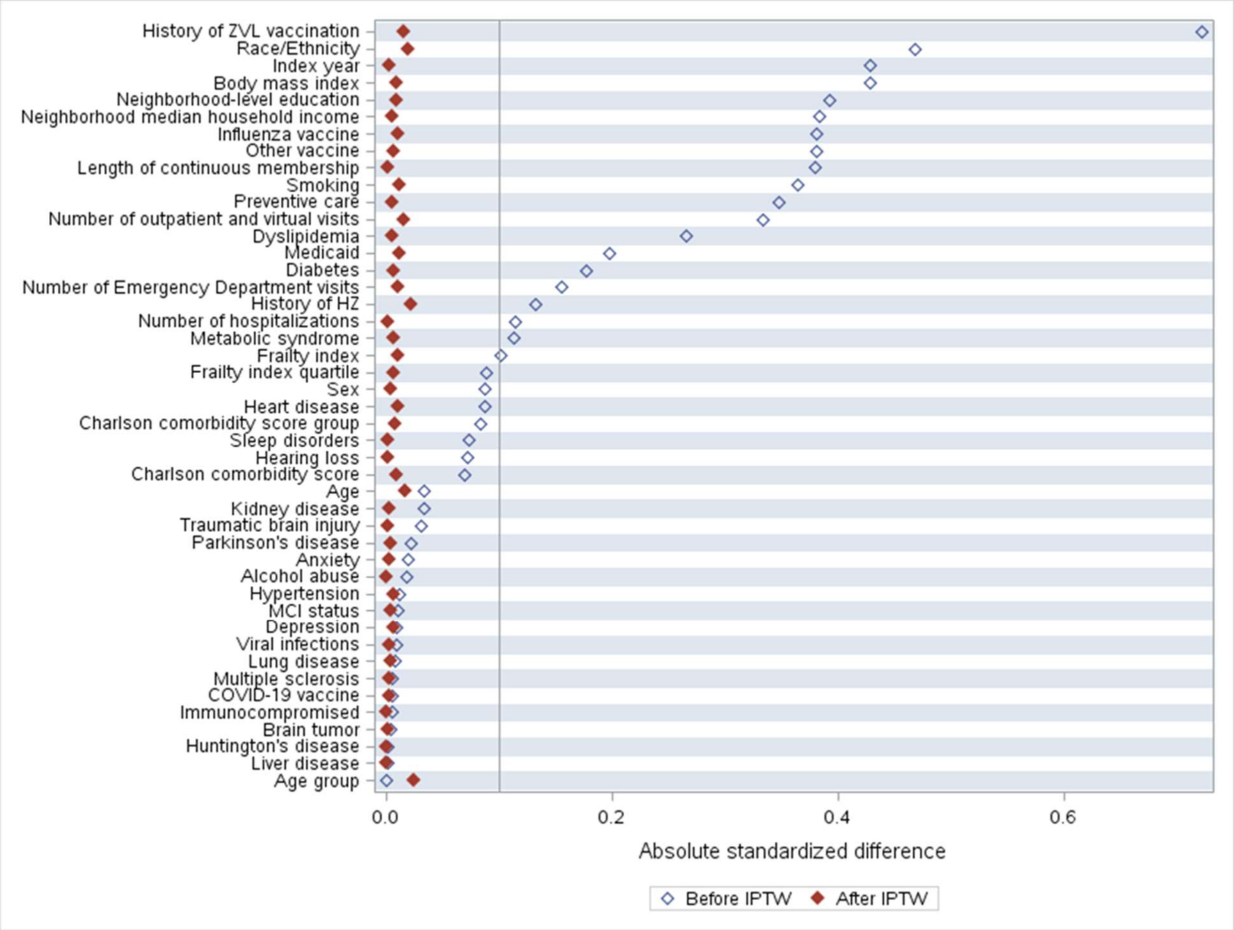

HZ, herpes zoster; IPTW, inverse probability of treatment weighting; MCI, mild cognitive impairment; RZV, recombinant zoster vaccine; Tdap, tetanus, diphtheria, and acellular pertussis vaccine; ZVL, zoster vaccine live

Supplementary Table 6. Baseline cohort characteristics of the two-dose RZV-vaccinated and Tdap-vaccinated cohorts after weighting.

	RZV-vaccinated<br>n=65,800	Tdap-vaccinated<br>n=65,800	ASD
Age at index date, years			0.017
Mean (SD)	73.34 (6.32)	73.23 (6.29)	
Median	72	72	
Q1, Q3	68, 77	68, 77	
Minimum, Maximum	65, 101	65, 105	
Age at index date, years, n (%)			0.025
65–69	21,602 (32.8)	22,099 (33.6)	
70–74	20,281 (30.8)	20,475 (31.1)	
75–79	12,892 (19.6)	12,704 (19.3)	
≥80	11,025 (16.8)	10,521 (16.0)	
Sex, n (%)			0.004
Female	36,550 (55.5)	36,668 (55.7)	
Male	29,250 (44.5)	29,132 (44.3)	
Race/Ethnicity, n (%)			0.020
Non-Hispanic White	35,335 (53.7)	35,161 (53.4)	
Non-Hispanic Black	4601 (7.0)	4657 (7.1)	
Hispanic	15,141 (23.0)	15,403 (23.4)	
Non-Hispanic Asian	8826 (13.4)	8872 (13.5)	
Other/Multiple/Unknown	1897 (2.9)	1707 (2.6)	
History of ZVL vaccination[a], n (%)			0.016
No	31,182 (47.4)	31,535 (47.9)	
Yes, ≤5 years	7259 (11.0)	6965 (10.6)	
Yes, >5 years	27,359 (41.6)	27,300 (41.5)	
MCI status[b], n (%)			0.004
No	64,699 (98.3)	64,664 (98.3)	
Yes	1101 (1.7)	1136 (1.7)	
History of HZ[a], n (%)			0.023
No	56,831 (86.4)	56,550 (85.9)	
Yes, ≤2 years	2724 (4.1)	3031 (4.6)	
Yes, >2 years	6246 (9.5)	6219 (9.5)	
Length of continuous membership[a], years, n (%)			0.002
1–<5	12,718 (19.3)	12,698 (19.3)	
5–<10	10,883 (16.5)	10,929 (16.6)	
≥10	42,199 (64.1)	42,173 (64.1)	
Other vaccines[c], n (%)			
Influenza vaccine	56,093 (85.2)	55,852 (84.9)	0.010
COVID-19 vaccine	1 (0.0)	2 (0.0)	0.002
Other[d]	16,572 (25.2)	16,750 (25.5)	0.006
Number of outpatient and virtual visits[c], n (%)			0.016
0–4	11,065 (16.8)	11,438 (17.4)	
5–10	21,057 (32.0)	20,733 (31.5)	

	RZV-vaccinated n=65,800	Tdap-vaccinated n=65,800	ASD
≥11	33,678 (51.2)	33,629 (51.1)	
Number of Emergency Department visits[c], n (%)			0.010
0	52,418 (79.7)	52,633 (80.0)	
1	8967 (13.6)	8911 (13.5)	
≥2	4415 (6.7)	4255 (6.5)	
Number of hospitalizations[c], n (%)			0.002
0	61,401 (93.3)	61,381 (93.3)	
1	3404 (5.2)	3427 (5.2)	
≥2	995 (1.5)	992 (1.5)	
Preventive care[c], n (%)	36,795 (55.9)	36,993 (56.2)	0.006
Comorbidities[c], n (%)			
Kidney disease	11,240 (17.1)	11,176 (17.0)	0.003
Heart disease	5677 (8.6)	5489 (8.3)	0.010
Lung disease	11,582 (17.6)	11,467 (17.4)	0.005
Liver disease	2910 (4.4)	2910 (4.4)	0.000
Diabetes	18,577 (28.2)	18,386 (27.9)	0.007
Brain tumor	36 (0.1)	33 (0.0)	0.002
Traumatic brain injury	258 (0.4)	265 (0.4)	0.002
Hearing loss	4600 (7.0)	4639 (7.1)	0.002
Parkinson’s disease	466 (0.7)	490 (0.7)	0.004
Huntington's disease	3 (0.0)	3 (0.0)	0.001
Multiple sclerosis	118 (0.2)	126 (0.2)	0.003
Anxiety	6925 (10.5)	6860 (10.4)	0.003
Depression	8579 (13.0)	8442 (12.8)	0.006
Sleep disorders	9012 (13.7)	8960 (13.6)	0.002
Metabolic syndrome	6877 (10.5)	6745 (10.3)	0.007
Hypertension	38,385 (58.3)	38,160 (58.0)	0.007
Alcohol abuse	952 (1.4)	945 (1.4)	0.001
Dyslipidemia	44,779 (68.1)	44,616 (67.8)	0.005
Viral infections	2812 (4.3)	2847 (4.3)	0.003
Immunocompromised at index date[e], n (%)	3524 (5.4)	3511 (5.3)	0.001
Charlson comorbidity score[c,f]			0.010
Mean (SD)	1.78 (2.05)	1.75 (2.03)	
Median	1	1	
Q1, Q3	0, 3	0, 3	
Minimum, Maximum	0, 17	0, 17	
Charlson comorbidity score[c,f], n (%)			0.008
0	22,846 (34.7)	23,080 (35.1)	
1	14,564 (22.1)	14,561 (22.1)	
≥2	28,390 (43.1)	28,158 (42.8)	
Frailty index[c,g]			0.011
Mean (SD)	0.13 (0.04)	0.13 (0.04)	
Median	0.13	0.13	

	RZV-vaccinated<br>n=65,800	Tdap-vaccinated<br>n=65,800	ASD
Q1, Q3	0.11, 0.15	0.11, 0.15	
Minimum, Maximum	0.04, 0.37	0.05, 0.37	
Frailty index[c,g], n (%)			0.006
Q1	16,225 (24.7)	16,224 (24.7)	
Q2	16,268 (24.7)	16,429 (25.0)	
Q3	16,480 (25.0)	16,366 (24.9)	
Q4, most frail	16,827 (25.6)	16,782 (25.5)	
Smoking[c], n (%)			0.012
No	45,101 (68.5)	45,064 (68.5)	
Yes	16,385 (24.9)	16,242 (24.7)	
Unknown	4314 (6.6)	4494 (6.8)	
Body mass index[h], n (%)			0.009
<18.5	803 (1.2)	799 (1.2)	
18.5–<25	18,332 (27.9)	18,170 (27.6)	
25–<30	22,939 (34.9)	23,031 (35.0)	
≥30	18,933 (28.8)	18,883 (28.7)	
Unknown	4793 (7.3)	4918 (7.5)	
Neighborhood median household income, n (%)			0.006
<$40,000	3347 (5.1)	3306 (5.0)	
$40,000–$59,999	11,438 (17.4)	11,417 (17.4)	
$60,000–$79,999	14,638 (22.2)	14,761 (22.4)	
≥$80,000	36,306 (55.2)	36,239 (55.1)	
Unknown	71 (0.1)	77 (0.1)	
Neighborhood-level education[i], n (%)			0.009
≤High school	14,450 (22.0)	14,691 (22.3)	
>High school	51,282 (77.9)	51,035 (77.6)	
Unknown	69 (0.1)	75 (0.1)	
Medicaid, n (%)	3311 (5.0)	3139 (4.8)	0.012
Year of index date, n (%)			0.003
2018	12,798 (19.5)	12,771 (19.4)	
2019	29,383 (44.7)	29,301 (44.5)	
2020	23,619 (35.9)	23,727 (36.1)	

[a]Defined based on all available medical records prior to the index date.
[b]Defined based on all available medical records prior to the index date to 6 months after the index date.
[c]Defined in the year prior to the index date.
[d]Among subjects who received other vaccines in the year prior to the index date: pneumococcal (64.7%), Tdap/Td (23.4%), hepatitis A or B (12.2%), and other vaccines (13.9%).
[e]Immunocompromised defined as HIV/AIDS, hematopoietic stem cell/solid organ transplant, leukemia/lymphoma, congenital and other immunodeficiencies, or asplenia/hyposplenia at any time prior to the index date, or immunosuppressive medication at index date.
[f]Possible range: 0–29.[1]
[g]Possible range: 0–1.[2]
[h]Defined as most recent in the year prior to the index date.
[i]Defined as <50% or ≥50% of the neighborhood that attained more than high school education.

AIDS, acquired immunodeficiency syndrome; ASD, absolute standardized difference; HIV, human immunodeficiency virus; HZ, herpes zoster; MCI, mild cognitive impairment; n, number; Q, quartile; RZV, recombinant zoster vaccine; SD, standard

deviation; Td, tetanus and diphtheria vaccine; Tdap, tetanus, diphtheria, and acellular pertussis vaccine; ZVL, zoster vaccine live

Supplementary Table 7. Incidence rate and hazard ratio of dementia among RZV-vaccinated versus unvaccinated or Tdap-vaccinated individuals using Cox regression model with IPTW.

Comparison Groups	RZV-vaccinated				Comparison Group				Hazard Ratio (95% CI)	
	n	Number of cases	Number of person-years	Incidence per 1000 person-years (95% CI)	n	Number of cases	Number of person-years	Incidence per 1000 person-years (95% CI)	Unadjusted	Adjusted[a]
Unvaccinated	65,800	2401	223,576.46	10.74 (10.32–11.18)	263,200	10,983	476,623.82	23.04 (22.61–23.48)	0.45 (0.43–0.47)	0.49 (0.46–0.51)
Tdap-vaccinated					65,800	2835	156,676.64	18.09 (17.44–18.77)	0.56 (0.53–0.59)	0.73 (0.67–0.79)

[a]Adjusted for time-varying vaccination status (influenza, COVID-19, and other vaccines) besides IPTW.

CI, confidence interval; IPTW, inverse probability of treatment weighting; n, number; RZV, recombinant zoster vaccine; Tdap, tetanus, diphtheria, and acellular pertussis vaccine

Supplementary Table 8. Incidence rate and hazard ratio of NCO in two-dose RZV-vaccinated versus unvaccinated individuals using Cox regression model with IPTW.

Outcomes	Vaccinated				Unvaccinated				Hazard Ratio (95% CI)	
	n	Number of cases	Number of person years	Incidence per 1000 person-years (95% CI)	n	Number of cases	Number of person years	Incidence per 1000 person-years (95% CI)	Unadjusted	Adjusted[a]
Any NCO from below	65,800	2171	222,777.21	9.75 (9.34–10.16)	263,200	4877	484,826.66	10.06 (9.78–10.35)	0.97 (0.92–1.02)	0.94 (0.89–1.00)[d]
Wrist fracture[b]	65,800	616	225,530.11	2.73 (2.52–2.96)	263,200	1107	489,343.01	2.26 (2.13–2.40)	1.20 (1.08–1.32)	1.15 (1.03–1.29)
Acute pancreatitis[c]	65,800	383	226,038.31	1.69 (1.53–1.87)	263,200	1144	489,521.92	2.34 (2.21–2.48)	0.72 (0.64–0.81)	0.75 (0.66–0.85)
Appendicitis[c]	65,800	199	226,273.03	0.88 (0.77–1.01)	263,200	442	490,198.63	0.90 (0.82–0.99)	1.00 (0.85–1.18)	0.98 (0.81–1.18)
Acute cholecystitis[c]	65,800	170	226,383.31	0.75 (0.65–0.87)	263,200	545	490,180.29	1.11 (1.02–1.21)	0.66 (0.56–0.79)	0.70 (0.58–0.86)
Adhesive capsulitis of the shoulder[c]	65,800	675	225,359.02	3.00 (2.78–3.23)	263,200	1314	489,178.00	2.69 (2.54–2.84)	1.13 (1.03–1.25)	1.05 (0.94–1.17)
Trigeminal neuralgia[c]	65,800	189	226,282.16	0.84 (0.72–0.96)	263,200	447	490,230.66	0.91 (0.83–1.00)	0.91 (0.77–1.08)	0.84 (0.70–1.01)

[a]Adjusted for time-varying vaccination status (influenza, COVID-19, and other vaccines) besides IPTW.
[b]Wrist fracture recommended as NCO in Salmon, D.A., et al. (2023).[3]
[c]Composite NCO adapted from Taquet, M., et al. (2024).[4]
[d]Upper bound of the confidence interval is 0.998.

CI, confidence interval; IPTW, inverse probability of treatment weighting; n, number; NCO, negative control outcome; RZV, recombinant zoster vaccine

Supplementary Table 9. Baseline characteristics of two-dose RZV-vaccinated and unvaccinated cohorts for MCI analysis before weighting.

	RZV-Vaccinated n=64,744	Unvaccinated n=258,199	ASD
Age at index date, years			0.014
Mean (SD)	73.16 (6.10)	73.25 (6.64)	
Median	72	72	
Q1, Q3	68, 77	68, 77	
Minimum, Maximum	65, 101	65, 109	
Age at index date, years, n (%)			0.005
65–69	21,087 (32.6)	84,393 (32.7)	
70–74	20,503 (31.7)	81,941 (31.7)	
75–79	12,898 (19.9)	51,379 (19.9)	
≥80	10,256 (15.8)	40,486 (15.7)	
Sex, n (%)			0.002
Female	37,408 (57.8)	148,962 (57.7)	
Male	27,336 (42.2)	109,237 (42.3)	
Race/Ethnicity, n (%)			0.001
Non-Hispanic White	39,598 (61.2)	157,851 (61.1)	
Non-Hispanic Black	2949 (4.6)	11,733 (4.5)	
Hispanic	10,221 (15.8)	40,798 (15.8)	
Non-Hispanic Asian	10,393 (16.1)	41,509 (16.1)	
Other/Multiple/Unknown	1583 (2.4)	6308 (2.4)	
History of ZVL vaccination[a], n (%)			0.002
No	21,796 (33.7)	87,160 (33.8)	
Yes, ≤5 years	5639 (8.7)	22,481 (8.7)	
Yes, >5 years	37,309 (57.6)	148,558 (57.5)	
MCI status[b], n (%)			0.000
No	64,744 (100.0)	258,199 (100.0)	
History of HZ[a], n (%)			0.145
No	55,163 (85.2)	226,724 (87.8)	
Yes, ≤2 years	3402 (5.3)	6378 (2.5)	
Yes, >2 years	6179 (9.5)	25,097 (9.7)	
Length of continuous membership[a], years, n (%)			0.103
1–<5	7974 (12.3)	39,614 (15.3)	
5–<10	10,838 (16.7)	46,737 (18.1)	
≥10	45,932 (70.9)	171,848 (66.6)	
Other vaccines[c], n (%)			
Influenza vaccine	59,054 (91.2)	205,412 (79.6)	0.335
COVID-19 vaccine	1 (0.0)	21 (0.0)	0.01
Other[d]	20,976 (32.4)	56,713 (22.0)	0.236
Number of outpatient and virtual visits[c], n (%)			0.310
0–4	7506 (11.6)	59,695 (23.1)	
5–10	22,193 (34.3)	80,588 (31.2)	
≥11	35,045 (54.1)	117,916 (45.7)	
Number of Emergency Department visits[c], n (%)			0.095

	RZV-Vaccinated n=64,744	Unvaccinated n=258,199	ASD
0	53,756 (83.0)	205,895 (79.7)	
1	7939 (12.3)	35,321 (13.7)	
≥2	3049 (4.7)	16,983 (6.6)	
Number of hospitalizations[c], n (%)			0.097
0	61,378 (94.8)	239,240 (92.7)	
1	2729 (4.2)	14,040 (5.4)	
≥2	637 (1.0)	4919 (1.9)	
Preventive care[c], n (%)	41,641 (64.3)	137,614 (53.3)	0.225
Comorbidities[c], n (%)			
Kidney disease	10,361 (16.0)	44,724 (17.3)	0.035
Heart disease	4493 (6.9)	23,033 (8.9)	0.073
Lung disease	11,058 (17.1)	44,915 (17.4)	0.008
Liver disease	2750 (4.2)	10,942 (4.2)	0.001
Diabetes	15,462 (23.9)	70,158 (27.2)	0.076
Brain tumor	34 (0.1)	246 (0.1)	0.016
Traumatic brain injury	178 (0.3)	1105 (0.4)	0.026
Hearing loss	4984 (7.7)	16,616 (6.4)	0.049
Parkinson’s disease	385 (0.6)	1980 (0.8)	0.021
Huntington's disease	2 (0.0)	10 (0.0)	0.001
Multiple sclerosis	122 (0.2)	473 (0.2)	0.001
Anxiety	6740 (10.4)	27,135 (10.5)	0.003
Depression	8091 (12.5)	34,151 (13.2)	0.022
Sleep disorders	9252 (14.3)	33,177 (12.8)	0.042
Metabolic syndrome	5444 (8.4)	25,396 (9.8)	0.050
Hypertension	37,448 (57.8)	145,877 (56.5)	0.027
Alcohol abuse	834 (1.3)	3949 (1.5)	0.021
Dyslipidemia	47,412 (73.2)	167,228 (64.8)	0.184
Viral infections	2815 (4.3)	9806 (3.8)	0.028
Immunocompromised at index date[e], n (%)	3359 (5.2)	12,137 (4.7)	0.023
Charlson comorbidity score[c,f]			0.055
Mean (SD)	1.65 (1.92)	1.76 (2.09)	
Median	1	1	
Q1, Q3	0, 2	0, 3	
Minimum, Maximum	0, 17	0, 17	
Charlson comorbidity score[c,f], n (%)			0.081
0	22,734 (35.1)	95,504 (37.0)	
1	15,589 (24.1)	53,528 (20.7)	
≥2	26,421 (40.8)	109,167 (42.3)	
Frailty index[c,g]			0.092
Mean (SD)	0.13 (0.03)	0.13 (0.04)	
Median	0.13	0.13	
Q1, Q3	0.11, 0.15	0.11, 0.15	
Minimum, Maximum	0.04, 0.37	0.05, 0.40	
Frailty index[c,g], n (%)			0.093

	RZV-Vaccinated n=64,744	Unvaccinated n=258,199	ASD
Q1	15,940 (24.6)	65,907 (25.5)	
Q2	17,832 (27.5)	63,840 (24.7)	
Q3	16,963 (26.2)	64,141 (24.8)	
Q4, most frail	14,009 (21.6)	64,311 (24.9)	
Smoking[c], n (%)			0.307
No	47,383 (73.2)	168,394 (65.2)	
Yes	15,739 (24.3)	65,079 (25.2)	
Unknown	1622 (2.5)	24,726 (9.6)	
Body mass index[h], n (%)			0.318
<18.5	869 (1.3)	3757 (1.5)	
18.5–<25	21,109 (32.6)	69,670 (27.0)	
25–<30	23,883 (36.9)	85,780 (33.2)	
≥30	17,117 (26.4)	73,213 (28.4)	
Unknown	1766 (2.7)	25,779 (10.0)	
Neighborhood median household income, n (%)			0.189
<$40,000	2009 (3.1)	11,938 (4.6)	
$40,000–$59,999	8388 (13.0)	44,413 (17.2)	
$60,000–$79,999	12,983 (20.1)	58,322 (22.6)	
≥$80,000	41,307 (63.8)	142,778 (55.3)	
Unknown	57 (0.1)	748 (0.3)	
Neighborhood-level education[i], n (%)			0.169
≤High school	9363 (14.5)	53,091 (20.6)	
>High school	55,326 (85.5)	204,370 (79.2)	
Unknown	55 (0.1)	738 (0.3)	
Medicaid, n (%)	1748 (2.7)	9949 (3.9)	0.065
Year of index date, n (%)			0.004
2018	8033 (12.4)	31,729 (12.3)	
2019	34,363 (53.1)	136,999 (53.1)	
2020	22,348 (34.5)	89,471 (34.7)	
Years of follow-up[j]			N/A
Mean (SD)	3.41 (0.98)	1.85 (1.28)	
Median	3.60	1.95	
Q1, Q3	2.90, 4.02	0.56, 2.81	
Minimum, Maximum	0.00, 5.22	0.00, 5.05	
Individuals appear in both exposure groups, n (%)	25,538 (39.4)	25,538 (9.9)	N/A

[a]Defined based on all available medical records prior to the index date.
[b]Defined based on all available medical records prior to index date to 6 months after the index date.
[c]Defined in the year prior to the index date.
[d]Among subjects who received other vaccines in the year prior to the index date: pneumococcal (60.5%), Tdap/Td (31.6%), hepatitis A or B (10.1%), and other vaccine (12.9%).
[e]Immunocompromised defined as HIV/AIDS, hematopoietic stem cell/solid organ transplant, leukemia/lymphoma, congenital and other immunodeficiencies, or asplenia/hyposplenia at any time prior to the index date, or immunosuppressive medication at index date.
[f]Possible range: 0–29.[1]
[g]Possible range: 0–1.[2]
[h]Defined as most recent in the year prior to the index date.
[i]Defined as <50% or ≥50% of the neighborhood that attained more than high school education.

[j]Follow-up started from 6 months after the index date until the end of the follow-up period (31 December 2023), death, receipt of a dose of zoster vaccine, termination of KPSC membership (allowing for a 31-day gap in membership), or outcome of interest, whichever came first. Follow-up for MCI shown above.

AIDS, acquired immunodeficiency syndrome; ASD, absolute standardized difference; HIV, human immunodeficiency virus; HZ, herpes zoster; KPSC, Kaiser Permanente Southern California; MCI, mild cognitive impairment; n, number; N/A, not applicable; Q, quartile; RZV, recombinant zoster vaccine; SD, standard deviation; Td, tetanus and diphtheria vaccine; Tdap, tetanus, diphtheria, and acellular pertussis vaccine; ZVL, zoster vaccine live

Supplementary Table 10. Baseline characteristics of two-dose RZV-vaccinated and unvaccinated cohorts for MCI analysis after weighting.

	RZV-Vaccinated n=64,744	Unvaccinated n=258,199	ASD
Age at index date, years			0.018
Mean (SD)	73.34 (6.14)	73.23 (6.63)	
Median	72	72	
Q1, Q3	69, 77	68, 77	
Minimum, Maximum	65, 101	65, 109	
Age at index date, years, n (%)			0.033
65–69	20,408 (31.5)	84,757 (32.8)	
70–74	20,560 (31.8)	81,772 (31.7)	
75–79	13,078 (20.2)	51,349 (19.9)	
≥80	10,698 (16.5)	40,320 (15.6)	
Sex, n (%)			0.003
Female	37,267 (57.6)	148,955 (57.7)	
Male	27,477 (42.4)	109,244 (42.3)	
Race/Ethnicity, n (%)			0.011
Non-Hispanic White	39,447 (60.9)	157,816 (61.1)	
Non-Hispanic Black	3003 (4.6)	11,753 (4.6)	
Hispanic	10,174 (15.7)	40,778 (15.8)	
Non-Hispanic Asian	10,605 (16.4)	41,552 (16.1)	
Other/Multiple/Unknown	1515 (2.3)	6300 (2.4)	
History of ZVL vaccination[a], n (%)			0.045
No	20,467 (31.6)	86,911 (33.7)	
Yes, ≤5 years	6015 (9.3)	22,545 (8.7)	
Yes, >5 years	38,262 (59.1)	148,743 (57.6)	
MCI status[b], n (%)			0.000
No	64,744 (100.0)	258,199 (100.0)	
History of HZ[a], n (%)			0.008
No	56,390 (87.1)	225,334 (87.3)	
Yes, ≤2 years	2056 (3.2)	7856 (3.0)	
Yes, >2 years	6299 (9.7)	25,009 (9.7)	
Length of continuous membership[a], years, n (%)			0.011
1–<5	9413 (14.5)	38,048 (14.7)	
5–<10	11,327 (17.5)	46,006 (17.8)	
≥10	44,004 (68.0)	174,145 (67.4)	
Other vaccines[c], n (%)			
Influenza vaccine	54,382 (84.0)	211,515 (81.9)	0.055
COVID-19 vaccine	2 (0.0)	18 (0.0)	0.006
Other[d]	16,603 (25.6)	62,249 (24.1)	0.036
Number of outpatient and virtual visits[c], n (%)			0.059
0–4	11,948 (18.5)	53,638 (20.8)	
5–10	21,004 (32.4)	82,116 (31.8)	
≥11	31,792 (49.1)	122,445 (47.4)	
Number of Emergency Department visits[c], n (%)			0.014

	RZV-Vaccinated n=64,744	Unvaccinated n=258,199	ASD
0	51,704 (79.9)	207,577 (80.4)	
1	8851 (13.7)	34,608 (13.4)	
≥2	4189 (6.5)	16,014 (6.2)	
Number of hospitalizations[c], n (%)			0.004
0	60,196 (93.0)	240,348 (93.1)	
1	3413 (5.3)	13,410 (5.2)	
≥2	1135 (1.8)	4442 (1.7)	
Preventive care[c], n (%)	36,950 (57.1)	143,406 (55.5)	0.031
Comorbidities[c], n (%)			
Kidney disease	11,448 (17.7)	44,058 (17.1)	0.016
Heart disease	5727 (8.8)	22,023 (8.5)	0.011
Lung disease	11,602 (17.9)	44,779 (17.3)	0.015
Liver disease	2832 (4.4)	10,983 (4.3)	0.006
Diabetes	17,789 (27.5)	68,475 (26.5)	0.022
Brain tumor	48 (0.1)	223 (0.1)	0.004
Traumatic brain injury	284 (0.4)	1024 (0.4)	0.007
Hearing loss	4512 (7.0)	17,269 (6.7)	0.011
Parkinson’s disease	472 (0.7)	1895 (0.7)	0.001
Huntington's disease	2 (0.0)	11 (0.0)	0.002
Multiple sclerosis	122 (0.2)	475 (0.2)	0.001
Anxiety	6938 (10.7)	27,134 (10.5)	0.007
Depression	8662 (13.4)	33,786 (13.1)	0.009
Sleep disorders	8806 (13.6)	33,987 (13.2)	0.013
Metabolic syndrome	6417 (9.9)	24,662 (9.6)	0.012
Hypertension	38,042 (58.8)	146,656 (56.8)	0.040
Alcohol abuse	953 (1.5)	3823 (1.5)	0.001
Dyslipidemia	44,541 (68.8)	171,762 (66.5)	0.049
Viral infections	2555 (3.9)	10,089 (3.9)	0.002
Immunocompromised at index date[e], n (%)	3152 (4.9)	12,391 (4.8)	0.003
Charlson comorbidity score[c,f]			0.017
Mean (SD)	1.78 (2.03)	1.74 (2.06)	
Median	1	1	
Q1, Q3	0, 3	0, 3	
Minimum, Maximum	0, 17	0, 17	
Charlson comorbidity score[c,f], n (%)			0.043
0	22,349 (34.5)	94,435 (36.6)	
1	14,204 (21.9)	55,280 (21.4)	
≥2	28,190 (43.5)	108,485 (42.0)	
Frailty index[c,g]			0.009
Mean (SD)	0.13 (0.04)	0.13 (0.04)	
Median	0.13	0.13	
Q1, Q3	0.11, 0.15	0.11, 0.15	
Minimum, Maximum	0.04, 0.37	0.05, 0.40	
Frailty index[c,g], n (%)			0.041

	RZV-Vaccinated n=64,744	Unvaccinated n=258,199	ASD
Q1	15,255 (23.6)	65,387 (25.3)	
Q2	16,645 (25.7)	65,311 (25.3)	
Q3	16,643 (25.7)	64,852 (25.1)	
Q4, most frail	16,202 (25.0)	62,648 (24.3)	
Smoking[c], n (%)			0.077
No	44,218 (68.3)	172,541 (66.8)	
Yes	16,534 (25.5)	64,639 (25.0)	
Unknown	3992 (6.2)	21,019 (8.1)	
Body mass index[h], n (%)			0.082
<18.5	902 (1.4)	3692 (1.4)	
18.5–<25	18,635 (28.8)	72,585 (28.1)	
25–<30	22,525 (34.8)	87,715 (34.0)	
≥30	18,564 (28.7)	72,237 (28.0)	
Unknown	4119 (6.4)	21,970 (8.5)	
Neighborhood median household income, n (%)			0.003
<$40,000	2774 (4.3)	11,146 (4.3)	
$40,000–$59,999	10,543 (16.3)	42,194 (16.3)	
$60,000–$79,999	14,290 (22.1)	56,996 (22.1)	
≥$80,000	36,981 (57.1)	147,221 (57.0)	
Unknown	155 (0.2)	642 (0.2)	
Neighborhood-level education[i], n (%)			0.008
≤High school	12,314 (19.0)	49,915 (19.3)	
>High school	52,276 (80.7)	207,651 (80.4)	
Unknown	154 (0.2)	633 (0.2)	
Medicaid, n (%)	2420 (3.7)	9367 (3.6)	0.006
Year of index date, n (%)			0.033
2018	8447 (13.0)	31,819 (12.3)	
2019	34,813 (53.8)	137,083 (53.1)	
2020	21,483 (33.2)	89,297 (34.6)	

[a]Defined based on all available medical records prior to the index date.
[b]Defined based on all available medical records prior to index date to 6 months after the index date.
[c]Defined in the year prior to the index date.
[d]Among subjects who received other vaccines in the year prior to the index date: pneumococcal (60.5%), Tdap/Td (31.6%), hepatitis A or B (10.1%), and other vaccine (12.9%).
[e]Immunocompromised defined as HIV/AIDS, hematopoietic stem cell/solid organ transplant, leukemia/lymphoma, congenital and other immunodeficiencies, or asplenia/hyposplenia at any time prior to the index date, or immunosuppressive medication at index date.
[f]Possible range: 0–29.[1]
[g]Possible range: 0–1.[2]
[h]Defined as most recent in the year prior to the index date.
[i]Defined as <50% or ≥50% of the neighborhood that attained more than high school education.

AIDS, acquired immunodeficiency syndrome; ASD, absolute standardized difference; HIV, human immunodeficiency virus; HZ, herpes zoster; MCI, mild cognitive impairment; n, number; Q, quartile; RZV, recombinant zoster vaccine; SD, standard deviation; Td, tetanus and diphtheria vaccine; Tdap, tetanus, diphtheria, and acellular pertussis vaccine; ZVL, zoster vaccine live

Supplementary Table 11. Incidence rate and hazard ratio of MCI in two-dose RZV-vaccinated versus unvaccinated individuals using Cox regression model with IPTW.

	Vaccinated				Unvaccinated				Hazard Ratio (95% CI)	
**Outcomes**	**n**	**Number of cases**	**Number of person-years**	**Incidence per 1000 person-years (95% CI)**	**n**	**Number of cases**	**Number of person-years**	**Incidence per 1000 person-years (95% CI)**	**Unadjusted**	**Adjusted[a]**
MCI	64,744	1557	220,787.88	7.05 (6.71–7.41)	258,199	3564	476,862.93	7.47 (7.23–7.72)	0.93 (0.87–0.98)	0.86 (0.80–0.92)
<3.5 years of follow-up	64,744	1365	200,411.28	6.81 (6.46–7.18)	258,199	3492	465,149.50	7.51 (7.26–7.76)	0.90 (0.85–0.96)	0.84 (0.78–0.90)
≥3.5 years of follow-up	36,067	192	20,376.59	9.42 (8.18–10.85)	30,321	72	11,713.37	6.15 (4.88–7.74)	1.26 (1.07–1.48)	1.12 (0.94–1.35)

[a]Adjusted for time-varying vaccination status (influenza, COVID-19, and other vaccines) besides IPTW.

CI, confidence interval; IPTW, inverse probability of treatment weighting; MCI, mild cognitive impairment; n, number; RZV, recombinant zoster vaccine

Supplementary Figure 3. Cumulative incidence of MCI among two-dose RZV-vaccinated versus unvaccinated individuals.

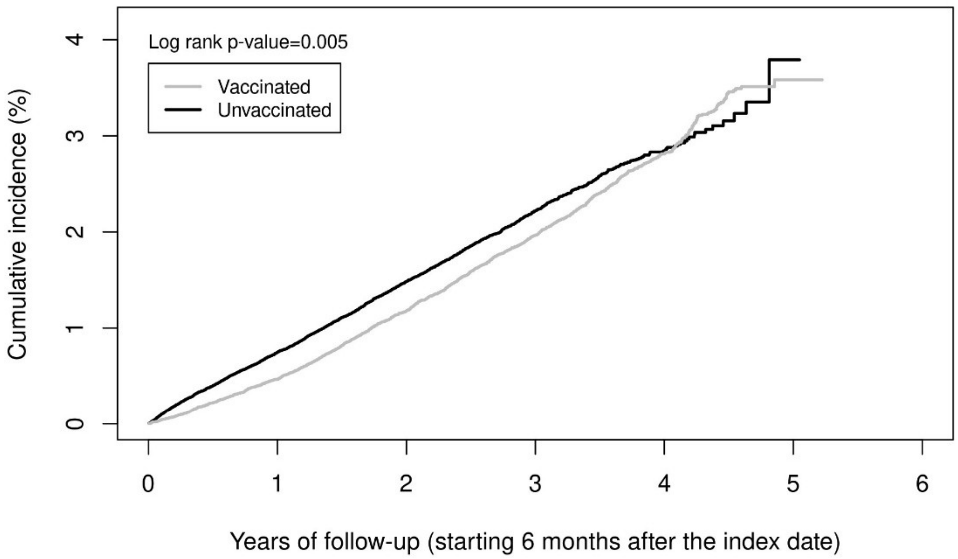

	0	1	2	3	4	5	6
**Vaccinated**							
Number at risk	64,744	61,998	59,170	47,435	16,932	151	0
Cumulative events	0	297	731	1,171	1,479	1,557	1,557
Cumulative incidence	0	0.47	1.18	1.97	2.81	3.58	3.58
**Unvaccinated**							
Number at risk	258,199	176,298	126,154	54,578	8,378	5	0
Cumulative events	0	1,537	2,663	3,332	3,547	3,564	3,564
Cumulative incidence	0	0.75	1.48	2.22	2.84	3.79	3.79

MCI, mild cognitive impairment; RZV, recombinant zoster vaccine

Supplementary Table 12. Time to diagnosis of dementia from MCI diagnosis.

Time, days	RZV-vaccinated[a] n=393	Unvaccinated[a] n=909
Mean (SD)	335.44 (297.12)	266.49 (274.92)
Median	261	193
Q1, Q3	98, 500	37, 396
Minimum, Maximum	0, 1506	0, 1577

[a]Among individuals diagnosed with incident MCI and then dementia during follow-up.

MCI, mild cognitive impairment; n, number; Q, quartile; RZV, recombinant zoster vaccine; SD, standard deviation

Supplementary Table 13. Baseline characteristics of two-dose RZV-vaccinated and unvaccinated cohort with index date prior to COVID-19 pandemic after weighting.

	RZV-vaccinated n=50,542	Unvaccinated n=202,168	ASD
Age at index date, years			0.007
Mean (SD)	73.61 (6.22)	73.56 (6.63)	
Median	72	72	
Q1, Q3	69, 77	68, 78	
Minimum, Maximum	65, 101	65, 109	
Age at index date, years, n (%)			0.036
65–69	15,079 (29.8)	63,151 (31.2)	
70–74	16,107 (31.9)	64,088 (31.7)	
75–79	10,463 (20.7)	41,375 (20.5)	
≥80	8894 (17.6)	33,554 (16.6)	
Sex, n (%)			0.005
Female	29,128 (57.6)	116,990 (57.9)	
Male	21,414 (42.4)	85,178 (42.1)	
Race/Ethnicity, n (%)			0.018
Non-Hispanic White	31,397 (62.1)	126,364 (62.5)	
Non-Hispanic Black	2212 (4.4)	8557 (4.2)	
Hispanic	7462 (14.8)	29,691 (14.7)	
Non-Hispanic Asian	8383 (16.6)	32,811 (16.2)	
Other/Multiple/Unknown	1088 (2.2)	4745 (2.3)	
History of ZVL vaccination[a], n (%)			0.041
No	14,919 (29.5)	63,296 (31.3)	
Yes, ≤5 years	4810 (9.5)	17,980 (8.9)	
Yes, >5 years	30,813 (61.0)	120,892 (59.8)	
MCI status[b], n (%)			0.007
No	49,603 (98.1)	198,231 (98.1)	
Yes	939 (1.9)	3937 (1.9)	
History of HZ[a], n (%)			0.004
No	44,045 (87.1)	176,033 (87.1)	
Yes, ≤2 years	1555 (3.1)	6365 (3.1)	
Yes, >2 years	4943 (9.8)	19,770 (9.8)	
Length of continuous membership[a], years, n (%)			0.026
1–<5	7186 (14.2)	26,951 (13.3)	
5–<10	8587 (17.0)	34,772 (17.2)	
≥10	34,769 (68.8)	140,446 (69.5)	
Other vaccines[c], n (%)			
Influenza vaccine	42,145 (83.4)	167,460 (82.8)	0.015
COVID-19 vaccine	0 (0)	0 (0)	0.000
Other[d]	12,222 (24.2)	49,089 (24.3)	0.002
Number of outpatient and virtual visits[c], n (%)			0.042
0–4	8574 (17.0)	37,543 (18.6)	
5–10	16,183 (32.0)	63,941 (31.6)	
≥11	25,785 (51.0)	100,684 (49.8)	

	RZV-vaccinated n=50,542	Unvaccinated n=202,168	ASD
Number of Emergency Department visits[c], n (%)			0.010
0	39,990 (79.1)	160,743 (79.5)	
1	7157 (14.2)	27,994 (13.8)	
≥2	3395 (6.7)	13,431 (6.6)	
Number of hospitalizations[c], n (%)			0.001
0	46,807 (92.6)	187,234 (92.6)	
1	2794 (5.5)	11,197 (5.5)	
≥2	941 (1.9)	3737 (1.8)	
Preventive care[c], n (%)	29,576 (58.5)	116,186 (57.5)	0.021
Comorbidities[c], n (%)			
Kidney disease	9153 (18.1)	36,265 (17.9)	0.005
Heart disease	4628 (9.2)	18,169 (9.0)	0.006
Lung disease	9342 (18.5)	36,797 (18.2)	0.007
Liver disease	2242 (4.4)	8781 (4.3)	0.005
Diabetes	13,750 (27.2)	54,112 (26.8)	0.010
Brain tumor	28 (0.1)	175 (0.1)	0.012
Traumatic brain injury	235 (0.5)	848 (0.4)	0.007
Hearing loss	3831 (7.6)	14,895 (7.4)	0.008
Parkinson’s disease	398 (0.8)	1655 (0.8)	0.003
Huntington's disease	2 (0.0)	12 (0.0)	0.004
Multiple sclerosis	89 (0.2)	373 (0.2)	0.002
Anxiety	5687 (11.3)	22,403 (11.1)	0.005
Depression	7067 (14.0)	28,135 (13.9)	0.002
Sleep disorders	7251 (14.3)	28,230 (14.0)	0.011
Metabolic syndrome	4925 (9.7)	19,579 (9.7)	0.002
Hypertension	30,152 (59.7)	118,462 (58.6)	0.022
Alcohol abuse	777 (1.5)	3152 (1.6)	0.002
Dyslipidemia	35,482 (70.2)	140,033 (69.3)	0.020
Viral infections	1999 (4.0)	7753 (3.8)	0.006
Immunocompromised at index date[e], n (%)	2481 (4.9)	9955 (4.9)	0.001
Charlson comorbidity score[c,f]			0.005
Mean (SD)	1.80 (2.04)	1.82 (2.09)	
Median	1	1	
Q1, Q3	0, 3	0, 3	
Minimum, Maximum	0, 17	0, 17	
Charlson comorbidity score[c,f], n (%)			0.022
0	16,980 (33.6)	69,993 (34.6)	
1	11,154 (22.1)	43,767 (21.6)	
≥2	22,408 (44.3)	88,408 (43.7)	
Frailty index[c,g]			0.005
Mean (SD)	0.14 (0.04)	0.14 (0.04)	
Median	0.13	0.13	
Q1, Q3	0.11, 0.16	0.11, 0.16	
Minimum, Maximum	0.04, 0.37	0.05, 0.40	

	RZV-vaccinated n=50,542	Unvaccinated n=202,168	ASD
Frailty index[c,g], n (%)			0.031
Q1	11,352 (22.5)	47,962 (23.7)	
Q2	12,748 (25.2)	49,865 (24.7)	
Q3	13,093 (25.9)	51,366 (25.4)	
Q4, most frail	13,348 (26.4)	52,975 (26.2)	
Smoking[c], n (%)			0.066
No	34,795 (68.8)	137,024 (67.8)	
Yes	13,264 (26.2)	52,124 (25.8)	
Unknown	2482 (4.9)	13,021 (6.4)	
Body mass index[h], n (%)			0.083
<18.5	760 (1.5)	3091 (1.5)	
18.5–<25	15,312 (30.3)	59,414 (29.4)	
25–<30	17,914 (35.4)	70,262 (34.8)	
≥30	14,253 (28.2)	56,404 (27.9)	
Unknown	2303 (4.6)	12,997 (6.4)	
Neighborhood median household income, n (%)			0.006
<$40,000	2296 (4.5)	9178 (4.5)	
$40,000–$59,999	8221 (16.3)	33,256 (16.4)	
$60,000–$79,999	11,211 (22.2)	44,547 (22.0)	
≥$80,000	28,675 (56.7)	114,642 (56.7)	
Unknown	139 (0.3)	545 (0.3)	
Neighborhood-level education[i], n (%)			0.010
≤High school	9209 (18.2)	37,614 (18.6)	
>High school	41,196 (81.5)	164,017 (81.1)	
Unknown	137 (0.3)	537 (0.3)	
Medicaid, n (%)	1901 (3.8)	6875 (3.4)	0.019
Year of index date, n (%)			0.043
2018	8517 (16.9)	32,587 (16.1)	
2019	35,191 (69.6)	139,380 (68.9)	
2020	6834 (13.5)	30,201 (14.9)	
Years of follow-up[j,k]			N/A
Mean (SD)	3.63 (0.97)	1.84 (1.37)	
Median	3.77	2.02	
Q1, Q3	3.47, 4.18	0.35, 2.96	
Minimum, Maximum	0.00, 5.22	0.00, 5.02	
Individuals appear in both exposure groups[k], n (%)	18,443 (36.5)	18,443 (9.1)	N/A

[a]Defined based on all available medical records prior to the index date.
[b]Defined based on all available medical records prior to index date to 6 months after the index date.
[c]Defined in the year prior to the index date.
[d]Among subjects who received other vaccines in the year prior to the index date: pneumococcal (61.3%), Tdap/Td (27.7%), hepatitis A or B (11.5%), and other vaccine (14.2%).
[e]Immunocompromised defined as HIV/AIDS, hematopoietic stem cell/solid organ transplant, leukemia/lymphoma, congenital and other immunodeficiencies, or asplenia/hyposplenia at any time prior to the index date, or immunosuppressive medication at index date.
[f]Possible range: 0–29.[1]
[g]Possible range: 0–1.[2]

[h]Defined as most recent in the year prior to the index date.
[i]Defined as <50% or ≥50% of the neighborhood that attained more than high school education.
[j]Follow-up started from 6 months after the index date until the end of the follow-up period (31 December 2023), death, receipt of a dose of zoster vaccine, termination of KPSC membership (allowing for a 31-day gap in membership), or outcome of interest, whichever came first. Follow-up for dementia (primary outcome) shown above.
[k]Unweighted statistics shown.

AIDS, acquired immunodeficiency syndrome; ASD, absolute standardized difference; HIV, human immunodeficiency virus; HZ, herpes zoster; KPSC, Kaiser Permanente Southern California; MCI, mild cognitive impairment; n, number; N/A, not applicable; Q, quartile; RZV, recombinant zoster vaccine; SD, standard deviation; Td, tetanus and diphtheria vaccine; Tdap, tetanus, diphtheria, and acellular pertussis vaccine; ZVL, zoster vaccine live

Supplementary Table 14. Incidence rate and hazard ratio of outcomes for sensitivity analyses among two-dose RZV-vaccinated versus unvaccinated individuals using Cox regression model with IPTW.

	Vaccinated				Unvaccinated				Hazard Ratio (95% CI)	
**Outcomes**	**n**	**Number of cases**	**Number of person-years**	**Incidence per 1000 person-years (95% CI)**	**n**	**Number of cases**	**Number of person-years**	**Incidence per 1000 person-years (95% CI)**	**Unadjusted**	**Adjusted[a]**
Dementia[b]	65,800	1757	224,236.60	7.84 (7.48–8.21)	263,200	7742	479,563.78	16.14 (15.79–16.51)	0.47 (0.45–0.50)	0.49 (0.46–0.52)
Dementia[c,d]	50,542	2012	183,296.80	10.98 (10.51–11.47)	202,168	9383	371,227.93	25.28 (24.77–25.80)	0.42 (0.40–0.44)	0.45 (0.43–0.48)
Dementia[d,e]	39,813	1540	140,636.02	10.95 (10.42–11.51)	159,252	9834	491,231.77	20.02 (19.62–20.42)	0.54 (0.52–0.57)	0.60 (0.56–0.63)

[a]Adjusted for time-varying vaccination status (influenza, COVID-19, and other vaccines) besides IPTW.
[b]≥2 diagnoses or ≥1 diagnosis with medication.
[c]Among individuals with index date prior to COVID-19 pandemic.
[d]For this sensitivity analysis, the definition of dementia from the main analysis was used (≥1 diagnosis code).
[e]Follow-up not censored at receipt of HZ vaccine.

CI, confidence interval; HZ, herpes zoster; IPTW, inverse probability of treatment weighting; n, number; RZV, recombinant zoster vaccine

Supplementary Table 15. Baseline characteristics of two-dose RZV-vaccinated and unvaccinated cohort without censoring receipt of HZ vaccines during follow-up after weighting.

	Vaccinated n=39,813	Unvaccinated n=159,252	ASD
Age at index date, years			0.015
Mean (SD)	73.01 (6.43)	72.91 (6.83)	
Median	72	71	
Q1, Q3	68, 77	67, 77	
Minimum, Maximum	65, 101	65, 108	
Age at index date, years, n (%)			0.041
65–69	14,313 (36.0)	60,104 (37.7)	
70–74	11,644 (29.2)	45,973 (28.9)	
75–79	7134 (17.9)	27,931 (17.5)	
≥80	6722 (16.9)	25,244 (15.9)	
Sex, n (%)			0.002
Female	22,242 (55.9)	89,129 (56.0)	
Male	17,571 (44.1)	70,123 (44.0)	
Race/Ethnicity, n (%)			0.017
Non-Hispanic White	22,039 (55.4)	88,744 (55.7)	
Non-Hispanic Black	2539 (6.4)	9989 (6.3)	
Hispanic	8239 (20.7)	32,890 (20.7)	
Non-Hispanic Asian	5902 (14.8)	22,944 (14.4)	
Other/Multiple/Unknown	1093 (2.7)	4684 (2.9)	
History of ZVL vaccination[a], n (%)			0.054
No	17,421 (43.8)	73,930 (46.4)	
Yes, ≤5 years	4533 (11.4)	16,855 (10.6)	
Yes, >5 years	17,859 (44.9)	68,467 (43.0)	
MCI status[b], n (%)			0.000
No	39,085 (98.2)	156,342 (98.2)	
Yes	728 (1.8)	2910 (1.8)	
History of HZ[a], n (%)			0.008
No	34,647 (87.0)	138,886 (87.2)	
Yes, ≤2 years	1351 (3.4)	5193 (3.3)	
Yes, >2 years	3815 (9.6)	15,174 (9.5)	
Length of continuous membership[a], years, n (%)			0.014
1–<5	6813 (17.1)	27,729 (17.4)	
5–<10	6999 (17.6)	28,596 (18.0)	
≥10	26,001 (65.3)	102,926 (64.6)	
Other vaccines[c], n (%)			
Influenza vaccine	32,943 (82.7)	127,750 (80.2)	0.065
COVID-19 vaccine	1 (0.0)	12 (0.0)	0.006
Other[d]	11,271 (28.3)	42,174 (26.5)	0.041
Number of outpatient and virtual visits[c], n (%)			0.065
0–4	7479 (18.8)	34,000 (21.3)	
5–10	12,819 (32.2)	50,238 (31.5)	
≥11	19,515 (49.0)	75,014 (47.1)	

	Vaccinated n=39,813	Unvaccinated n=159,252	ASD
Number of Emergency Department visits[c], n (%)			0.013
0	31,639 (79.5)	127,324 (80.0)	
1	5492 (13.8)	21,601 (13.6)	
≥2	2682 (6.7)	10,327 (6.5)	
Number of hospitalizations[c], n (%)			0.005
0	36,944 (92.8)	147,938 (92.9)	
1	2116 (5.3)	8387 (5.3)	
≥2	753 (1.9)	2927 (1.8)	
Preventive care[c], n (%)	22,461 (56.4)	87,210 (54.8)	0.033
Comorbidities[c], n (%)			
Kidney disease	7000 (17.6)	26,921 (16.9)	0.018
Heart disease	3611 (9.1)	13,788 (8.7)	0.015
Lung disease	6990 (17.6)	26,927 (16.9)	0.017
Liver disease	1800 (4.5)	6983 (4.4)	0.007
Diabetes	11,280 (28.3)	43,272 (27.2)	0.026
Brain tumor	31 (0.1)	135 (0.1)	0.003
Traumatic brain injury	178 (0.4)	641 (0.4)	0.007
Hearing loss	2821 (7.1)	10,839 (6.8)	0.011
Parkinson’s disease	303 (0.8)	1211 (0.8)	0.000
Huntington's disease	1 (0.0)	7 (0.0)	0.002
Multiple sclerosis	70 (0.2)	294 (0.2)	0.002
Anxiety	4283 (10.8)	16,743 (10.5)	0.008
Depression	5321 (13.4)	20,560 (12.9)	0.014
Sleep disorders	5445 (13.7)	20,932 (13.1)	0.016
Metabolic syndrome	4062 (10.2)	15,619 (9.8)	0.013
Hypertension	23,347 (58.6)	90,051 (56.5)	0.042
Alcohol abuse	611 (1.5)	2440 (1.5)	0.000
Dyslipidemia	27,105 (68.1)	104,405 (65.6)	0.054
Viral infections	1633 (4.1)	6490 (4.1)	0.001
Immunocompromised at index date[e], n (%)	1996 (5.0)	7870 (4.9)	0.003
Charlson comorbidity score[c,f]			0.020
Mean (SD)	1.78 (2.06)	1.74 (2.09)	
Median	1	1	
Q1, Q3	0, 3	0, 3	
Minimum, Maximum	0, 16	0, 17	
Charlson comorbidity score[c,f], n (%)			0.048
0	13,866 (34.8)	59,092 (37.1)	
1	8687 (21.8)	33,864 (21.3)	
≥2	17,260 (43.4)	66,297 (41.6)	
Frailty index[c,g]			0.012
Mean (SD)	0.13 (0.04)	0.13 (0.04)	
Median	0.13	0.13	
Q1, Q3	0.11, 0.15	0.11, 0.15	
Minimum, Maximum	0.04, 0.37	0.05, 0.38	

	Vaccinated n=39,813	Unvaccinated n=159,252	ASD
Frailty index[c,g], n (%)			0.045
Q1	9568 (24.0)	41,367 (26.0)	
Q2	10,119 (25.4)	39,769 (25.0)	
Q3	10,092 (25.3)	39,174 (24.6)	
Q4, most frail	10,034 (25.2)	38,942 (24.5)	
Smoking[c], n (%)			0.090
No	27,172 (68.2)	106,037 (66.6)	
Yes	10,193 (25.6)	39,703 (24.9)	
Unknown	2448 (6.1)	13,512 (8.5)	
Body mass index[h], n (%)			0.092
<18.5	531 (1.3)	2229 (1.4)	
18.5–<25	11,184 (28.1)	43,578 (27.4)	
25–<30	13,953 (35.0)	54,320 (34.1)	
≥30	11,618 (29.2)	45,158 (28.4)	
Unknown	2527 (6.3)	13,967 (8.8)	
Neighborhood median household income, n (%)			0.004
<$40,000	1954 (4.9)	7843 (4.9)	
$40,000–$59,999	6983 (17.5)	28,021 (17.6)	
$60,000–$79,999	9047 (22.7)	36,306 (22.8)	
≥$80,000	21,721 (54.6)	86,628 (54.4)	
Unknown	108 (0.3)	454 (0.3)	
Neighborhood-level education[i], n (%)			0.007
≤High school	8527 (21.4)	34,509 (21.7)	
>High school	31,179 (78.3)	124,296 (78.0)	
Unknown	106 (0.3)	447 (0.3)	
Medicaid, n (%)	1768 (4.4)	6742 (4.2)	0.010
Year of index date, n (%)			0.040
2018	8609 (21.6)	32,691 (20.5)	
2019	20,593 (51.7)	81,562 (51.2)	
2020	10,610 (26.7)	45,000 (28.3)	
Years of follow-up[j,k]			N/A
Mean (sd)	3.53 (1.05)	3.08 (1.41)	
Median	3.73	3.51	
Q1, Q3	3.00, 4.32	2.54, 4.14	
Min, Max	0.00, 5.22	0.00, 5.22	
Individuals appear in both exposure groups, n (%)	0 (0.0)	0 (0.0)	N/A

[a]Defined based on all available medical records prior to the index date.
[b]Defined based on all available medical records prior to index date to 6 months after the index date.
[c]Defined in the one year prior to the index date.
[d]Other vaccines included pneumococcal (62.7%), Tdap/Td (30.5%), hepatitis A or B (10.6%), and other vaccine (12.0%).
[e]Immunocompromised defined as HIV/AIDS, hematopoietic stem cell/solid organ transplant, leukemia/lymphoma, congenital and other immunodeficiencies, or asplenia/hyposplenia at any time prior to the index date, or immunosuppressive medication at index date.
[f]Possible range: 0-29.[1]
[g]Possible range: 0-1.[2]
[h]Defined as most recent in one year prior to the index date.
[i]Defined as <50% or ≥50% of neighborhood attained more than high school education.

[j]Follow-up not censored at receipt of HZ vaccine.
[k]Unweighted statistics shown.

AIDS, acquired immunodeficiency syndrome; ASD, absolute standardized difference; HIV, human immunodeficiency virus; HZ, herpes zoster; MCI, mild cognitive impairment; n, number; N/A, not applicable; Q, quartile; RZV, recombinant zoster vaccine; SD, standard deviation; Td, tetanus and diphtheria vaccine; Tdap, tetanus, diphtheria, and acellular pertussis vaccine; ZVL, zoster vaccine live

Supplementary Table 16. Incidence rate and hazard ratio of dementia with follow-up starting 1 year after index date among two-dose RZV-vaccinated versus unvaccinated or Tdap-vaccinated individuals using Cox regression model with IPTW.

Comparison Groups	RZV-vaccinated				Comparison Group				Hazard Ratio (95% CI)	
	n	Number of cases	Number of person-years	Incidence per 1000 person-years (95% CI)	n	Number of cases	Number of person-years	Incidence per 1000 person-years (95% CI)	Unadjusted	Adjusted[a]
Unvaccinated	64,430	2186	191,110.13	11.44 (10.97–11.93)	198,236	7066	366,611.22	19.27 (18.83–19.73)	0.58 (0.55–0.60)	0.60 (0.57–0.64)
Tdap-vaccinated					60,969	2367	125,038.18	18.93 (18.18–19.71)	0.57 (0.54–0.61)	0.73 (0.67–0.79)

[a]Adjusted for time-varying vaccination status (influenza, COVID-19, and other vaccines) besides IPTW.

CI, confidence interval; IPTW, inverse probability of treatment weighting; n, number; RZV, recombinant zoster vaccine, Tdap, tetanus, diphtheria, and acellular pertussis vaccine

Supplementary Figure 4. Cumulative incidence estimates of dementia among two-dose RZV-vaccinated versus unvaccinated individuals with follow-up starting at 1 year after index date.

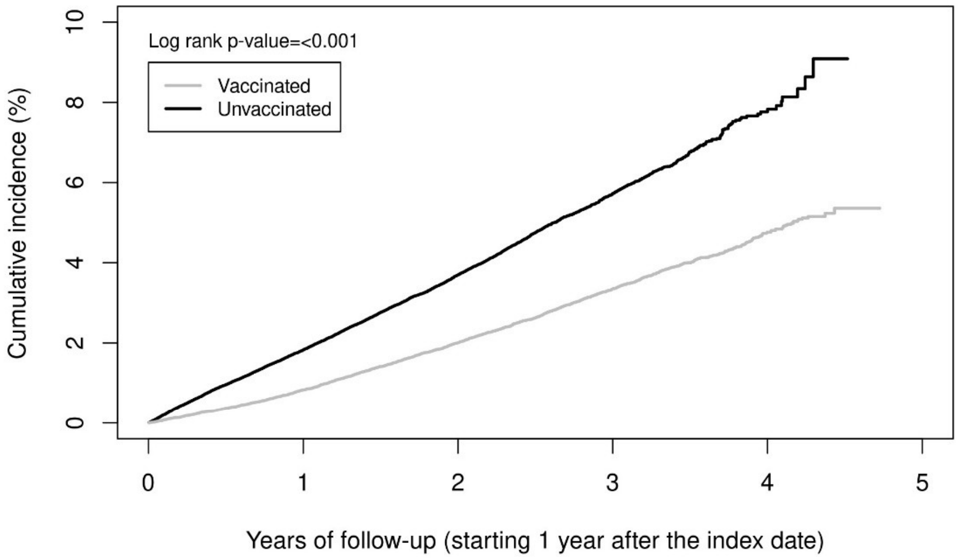

	0	1	2	3	4	5
**Vaccinated**						
Number at risk	64,430	61,438	58,415	36,395	6,582	0
Cumulative events	0	524	1,237	1,895	2,166	2,186
Cumulative incidence	0	0.83	2	3.35	4.76	5.36
**Unvaccinated**						
Number at risk	198,236	151,621	90,930	29,483	1,401	0
Cumulative events	0	3,257	5,630	6,819	7,060	7,066
Cumulative incidence	0	1.83	3.7	5.72	7.83	9.09

RZV, recombinant zoster vaccine

Supplementary Figure 5. Cumulative incidence estimates of dementia among two-dose RZV-vaccinated versus Tdap-vaccinated individuals with follow-up starting 1 year after index date.

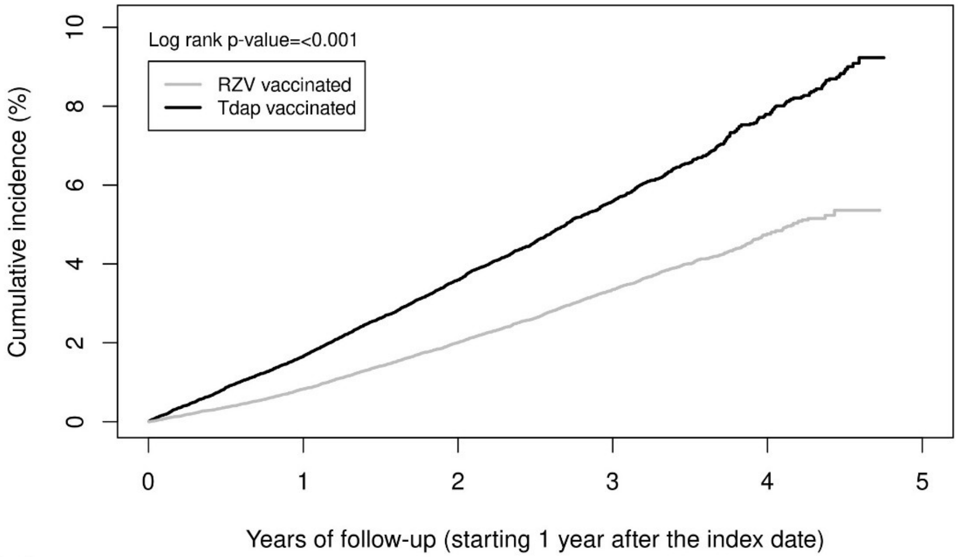

**RZV vaccinated**						
Number at risk	64,430	61,438	58,415	36,395	6,582	0
Cumulative events	0	524	1,237	1,895	2,166	2,186
Cumulative incidence	0	0.83	2	3.35	4.76	5.36
**Tdap vaccinated**						
Number at risk	60,969	47,084	30,878	15,151	3,571	0
Cumulative events	0	919	1,696	2,154	2,330	2,367
Cumulative incidence	0	1.65	3.59	5.59	7.79	9.23

RZV, recombinant zoster vaccine; Tdap, tetanus, diphtheria, and acellular pertussis vaccine

Supplementary Methods

Covariates

Covariates were identified from electronic health records and considered in analyses when feasible and appropriate as potential confounders or stratification variables (effect modifiers).

Covariates were identified at the following timepoints:

Prior to or including the index date or within 6 months of the index date:

- Mild cognitive impairment (MCI) status, based on the presence or absence of ≥1 International Classification of Diseases Version 9 (ICD-9) and/or ICD-10 code for MCI (**Supplementary Table 17**).

Prior to the index date:

- History of zoster vaccine live vaccination.
- History of herpes zoster (HZ) identified using ICD-9 and ICD-10 codes from hospital, outpatient (including virtual visits), and Emergency Department settings.
- Length of continuous membership.

In the year prior to the index date:

- Other vaccinations (e.g., influenza vaccine, COVID-19 vaccine, or other vaccines) received.
- Healthcare utilization (number of outpatient [including virtual visits]/Emergency Department/inpatient encounters) and preventive care.
- Brain tumor, comorbidities (kidney disease, heart disease, lung disease, liver disease, diabetes mellitus), Charlson comorbidity index, frailty index,[2] smoking history, body mass index (BMI), traumatic brain injury, hearing loss, Parkinson's disease, multiple sclerosis, Huntington's disease, mental health diagnoses (anxiety, depression), sleep disorders,

metabolic syndrome (by ICD-10 code for metabolic syndrome or composite of BMI ≥30, hypertension, and diabetes mellitus), hypertension, alcohol abuse, dyslipidemia, viral infections (e.g., influenza, SARS-CoV-2/COVID-19, etc.).

At index date:

- Demographic and socioeconomic variables: age, sex, race/ethnicity, income (neighborhood level census tract), education (neighborhood level census tract), Medicaid status.

During the baseline period (1 year prior to the index date for vaccinations and all available history prior to the index date for HZ status), within 6 months of the index date, and during follow-up period:

- Vaccinations (time-varying) during follow-up (e.g., receipt of at least one influenza vaccine, COVID-19 vaccine, or other vaccines).
- HZ status (time-varying) during follow-up determined by no HZ occurrence and any HZ history or occurrence of incident HZ during follow-up. History of HZ was defined by ICD-10 codes. Incident HZ during follow-up was defined by ICD-10 codes from virtual, outpatient, Emergency Department, and hospital settings with a non-topical antiviral prescription (acyclovir, valacyclovir, famciclovir) within 7 days before or after the date of HZ diagnosis. In addition, to ensure that HZ cases were incident, no non-topical (i.e., oral or parenteral) antiviral medication was allowed in the 183 days to 8 days prior to the date of HZ diagnosis.

Immunocompromised status

- HIV/AIDS, hematopoietic stem cell/solid organ transplant at any time prior to the index date using registry information; leukemia/lymphoma, congenital and other immunodeficiencies, asplenia/hyposplenia identified by ICD-10 code at any time prior to

the index date (from 01 October 2015 onwards); immunosuppressive medication at index date.

Details of statistical analyses

Inverse Probablity of Treatment Weighting (IPTW) approach

The weight for each vaccinated subject was equal to the inverse of the probability of receiving the vaccine and the weight for each unvaccinated subject was equal to the inverse of 1 minus the probability of receiving the vaccine (i.e., the probability of not receiving the vaccine). The weight was normalized (stabilized) by dividing by the average weight of each exposure group, as previously described.[5] Absolute standardized difference (ASD) based on the weighted study population was used to assess whether balance of covariates was achieved between vaccinated and unvaccinated groups. An ASD <0.10 was considered a negligible difference.

Death was considered a competing risk. Cause-specific hazards models in which the competing events are treated as censored observations were used.[6,7] If balance was not reached for some covariates among the weighted cohort (residual imbalance), the covariates in the weighted Cox regression model were further controlled. Vaccinations (receipt of influenza vaccine, COVID-19 vaccine, or other vaccines) during follow-up were adjusted for as time-varying covariates in the Cox regression model as well. By design, individuals could contribute person-time to both exposure groups but no repeated events were possible. Since more than 5% of participants were in both exposure groups, a possible correlation between subjects was accounted for using robust variance estimation.

In case of loss to follow-up, which could break the matched sets and possibly cause imbalance on matching/weighted covariates, a double-adjustment approach was applied, i.e., control for covariates included in the treatment model in the outcome models for IPTW analysis[8] to assess the robustness of the findings.

Details of sensitivity analyses

The alternative dementia definition included (1) two dementia diagnoses by ICD-10 code, with the second diagnosis occurring at least 7 days after but within 1 year of the first diagnosis code, as previously described,[9] or (2) ≥1 dementia diagnosis made by ICD-10 code with medication to treat dementia symptoms prescribed within 1 year of the first diagnosis code (**Supplementary Table 18)**. For these alternative outcome definitions, an additional 6 months of records (through 30 June 2024) were searched for the second dementia diagnosis or dementia medication.

Supplementary Table 17. Outcome definition ICD-10 codes.

Outcome	ICD-10 Code
Dementia	F01*, F02*, F03*, G30*, G31.0*, G31.1, G31.83
Vascular dementia	F01*
Dementia in other diseases classified elsewhere	F02*
Unspecified dementia	F03*
Alzheimer's disease	G30*
Frontotemporal dementia	G31.0*
Senile degeneration of the brain	G31.1
Neurocognitive disorder with Lewy bodies	G31.83
Mild Cognitive Impairment	G31.84; 331.83 (ICD-9)[a]
Composite negative control outcome[3,4]	
Wrist fracture	S62.001A, S62.001B, S62.002A, S62.002B, S62.009A, S62.009B, S62.011A, S62.011B, S62.012A, S62.012B, S62.013A, S62.013B, S62.014A, S62.014B, S62.015A, S62.015B, S62.016A, S62.016B, S62.021A, S62.021B, S62.022A, S62.022B, S62.023A, S62.023B, S62.024A, S62.024B, S62.025A, S62.025B, S62.026A, S62.026B, S62.031A, S62.031B, S62.032A, S62.032B, S62.033A, S62.033B, S62.034A, S62.034B, S62.035A, S62.035B, S62.036A, S62.036B, S62.101A, S62.101B, S62.102A, S62.102B, S62.109A, S62.109B, S62.111A, S62.111B, S62.112A, S62.112B, S62.113A, S62.113B, S62.114A, S62.114B, S62.115A, S62.115B, S62.116A, S62.116B, S62.121A, S62.121B, S62.122A, S62.122B, S62.123A, S62.123B, S62.124A, S62.124B, S62.125A, S62.125B, S62.126A, S62.126B, S62.131A, S62.131B, S62.132A, S62.132B, S62.133A, S62.133B, S62.134A, S62.134B, S62.135A, S62.135B, S62.136A, S62.136B, S62.141A, S62.141B, S62.142A, S62.142B, S62.143A, S62.143B, S62.144A, S62.144B, S62.145A, S62.145B, S62.146A, S62.146B, S62.151A, S62.151B, S62.152A, S62.152B, S62.153A, S62.153B, S62.154A, S62.154B, S62.155A, S62.155B, S62.156A, S62.156B, S62.161A, S62.161B, S62.162A, S62.162B, S62.163A, S62.163B, S62.164A, S62.164B, S62.165A, S62.165B, S62.166A, S62.166B, S62.171A, S62.171B, S62.172A, S62.172B, S62.173A, S62.173B, S62.174A, S62.174B, S62.175A, S62.175B, S62.176A, S62.176B, S62.181A, S62.181B, S62.182A, S62.182B, S62.183A, S62.183B, S62.184A, S62.184B, S62.185A, S62.185B, S62.186A, S62.186B, S62.90XA, S62.90XB, S62.91XA, S62.91XB, S62.92XA, S62.92XB
Acute pancreatitis	K85*
Appendicitis	K35*
Acute cholecystitis	K81.0
Adhesive capsulitis of the shoulder	M75.0*
Trigeminal neuralgia	G50.0

*All subcodes found below the one listed were included in the definition.
[a]All available patient MCI diagnoses, including those made with an ICD-9 code, were screened for primary analysis.

ICD, International Classification of Diseases; MCI, mild cognitive impairment

Supplementary Table 18. Medications for the management of dementia symptoms.

Generic Name	Medication National Drug Code
Lecanemab	62856021201, 62856021501
Aducanumab	64406010101, 64406010202
Donepezil	00093073805, 00093073856, 00093073898, 00093073905, 00093073956, 00093073998, 00093540765, 00093540865, 00143974709 ,00143974730, 00143974809, 00143974830, 00228452903, 00228452909, 00378102577, 00378102593, 00378514405, 00378514477, 00378514493, 00378514505, 00378514577, 00378514593, 00781527410, 00781527413, 00781527431, 00781527492, 00781527510, 00781527513, 00781527531, 00781527592, 00781527664, 00781527764, 00904624261, 00904624361, 00904635461, 00904635561, 00904640846, 00904640861, 00904640880, 00904640889, 00904640946, 00904640961, 00904640980, 00904640989, 00904647761, 00904647861, 12280029230, 12280029290, 12280036715, 12280036730, 12280036790, 13668010205, 13668010210, 13668010230, 13668010240, 13668010271, 13668010274, 13668010290, 13668010305, 13668010310, 13668010326, 13668010330, 13668010371, 13668010374, 13668010390, 16571077803, 16571077809, 16571077810, 16571077850, 16571077903, 16571077909, 16571077910, 16571077950, 24979000406, 24979000407, 29300025011, 29300025087, 29300025111, 29300025187, 31722013910, 31722014010, 31722073705, 31722073730, 31722073790, 31722073805, 31722073830, 31722073890, 33342002707, 33342002710, 33342002715, 33342002744, 33342002807, 33342002810, 33342002815, 33342002844, 33342002907, 33342002960, 33342003007, 33342003060, 33342006107, 33342006110, 42254030430, 42254030530, 42254030590, 42291024690, 42291025490, 42291025590, 42543070201, 42543070205, 42543070210, 42543070230, 42543070290, 42543070301, 42543070305, 42543070310, 42543070330, 42543070390, 42582031109, 42582031130, 42582031209, 42582031230, 43063050090, 43063065990, 43547027503, 43547027509, 43547027511, 43547027603, 43547027609, 43547027611, 43547038203, 43547038209, 45963056004, 45963056008, 45963056030, 45963056104, 45963056108, 45963056130, 49848000590, 49848000690, 49884023209, 49884023211, 49999075330, 49999075430, 49999075490, 50090262700, 50090291000, 50090632900, 50436997401, 50436997501, 51079013830, 51079013856, 51079013930, 51079013956, 51407032030, 52343008930, 52343008990, 52343008999, 52343009030, 52343009090, 52343009099, 54569631000, 54569631100, 54868395200, 54868424500, 54868620700, 54868620701, 54868620800, 55111030230, 55111030290, 55111035605, 55111035610, 55111035630, 55111035690, 55111035705, 55111035710, 55111035730, 55111035790, 55289015121, 55289015130, 58864088630, 58864089530, 59746032930, 59746032990, 59746033001, 59746033030, 59746033090, 59762024501, 59762024502, 59762024503, 59762024504, 59762024601, 59762024602, 59762024603, 59762024604, 59762025001, 59762025201, 60429032110, 60429032130, 60429032190, 60429032210, 60429032230, 60429032290, 60687017101, 60687017111, 60687018201, 60687018211, 60687029201, 60687029211, 60687030301, 60687030311, 62332009230, 62332009290, 62332009291, 62332009330, 62332009390, 62332009391, 62756044018, 62756044081, 62756044083, 62756044518, 62756044581, 62756044583, 62856024511, 62856024530, 62856024541, 62856024590, 62856024611, 62856024630, 62856024641, 62856024690, 62856024730, 62856024790, 62856083130, 62856083230, 63304012810, 63304012830, 63304012877, 63304012890, 63304012910, 63304012930, 63304012977, 63304012990, 63629111701, 63629111801, 63629363201, 63629848301, 63739064610, 63739065210, 63739065310, 63739066710, 63739066810, 63739067810, 64380090704, 64380090705, 64380090707, 64380090804, 64380090807, 64679031101, 64679031103, 64679031105, 64679031201, 64679031203, 64679031205, 65038005501, 65038005503, 65038005601, 65038005603, 65862032530, 65862032590, 65862032599, 65862032630, 65862032690, 65862032699, 67544009215, 67544009217,

Generic Name	Medication National Drug Code
	67544009288, 67544009289, 68084047701, 68084047711, 68084047801, 68084047811, 68084072501, 68084072511, 68084073401, 68084073411, 68180052706, 68180052709, 68382034606, 68382034706, 69150041503, 69150041509, 69150041510, 69150041603, 69150041609, 69150041610, 69452010813, 69452010819, 69452010830, 69452010913, 69452010919, 69452010930, 71093012701, 71093012703, 71093012705, 71093012706, 71093012801, 71093012803, 71093012805, 71093012806, 71205094800, 71205094830, 71205094855, 71205094860, 71205094890, 72189003130, 72189003160, 72189003190, 65038005510, 65038005610, 65038005504, 65038005604, 75929008603, 75929008703, 71335079201, 31722073801, 31722073701, 31722073731, 31722073831, 71335079202, 68071319809, 82009011905, 82009011910, 82009012005, 82009012010, 71335079203, 71209002004, 71335087602, 71209001904, 46708029510, 46708029530, 46708029590, 46708029610, 68382034601, 68382034677, 68382034716, 65841072106, 65841072116 ,65841072201, 65841074916, 65841075016, 62756019483, 00615831305, 00615831330, 00615831339, 65841072101, 68071152303, 62756019418, 68382034605, 68382034710, 68382034777, 29300024801, 50228013930, 70771132000, 70771132003, 68788820803, 68788820806, 68788820809, 55111035678, 46708029591, 65841072206, 65841075001, 65841075010, 71335206501, 71335206502, 71335206503, 33342002712, 33342002731, 33342002812, 33342002831, 33342002906, 33342002912, 33342003006, 33342003012, 33342006112, 71335041601, 62332009210, 71335058203, 62756019481, 68382034701, 46708029691, 65841072210, 65841072230, 65841074901, 65841074906, 70518045200, 65862032501, 63187040130, 71335209301, 71335209302, 71335209303, 69844003701, 69844003702, 69844003703, 69844003801, 69844003802, 69844003803, 71335087603, 65862032505, 46708029690, 65862032614, 71335041603, 46708029630, 65841072105, 65841072130, 65841072216, 70518166600, 65841074910, 65841074930, 71335202201, 71335202202, 71335202203, 71335090001, 71335090003, 00615795105, 00615795130, 00615795139, 50090353700, 55111035778, 29300024813, 29300024913, 55154788200, 55154788300, 70771132005, 70771132009, 71209001901, 71209001911, 71335090002, 61919069290, 70771132004, 29300024819, 29300024910, 29300024810, 29300024919, 29300024901, 70771132001, 50228014030, 71335041602, 71335058201, 71335087601, 64380090805, 68382052105, 68382052177, 71209002001, 65841075006, 65862032601, 68382052101, 43063065930, 65841072205, 65862032510, 65862032605, 72189026590, 50228014010, 43547038210, 43547038250, 55111030205, 55111030210, 71209002011, 72162213600, 72162213603, 72162213605, 72162213609, 72162213700, 72162213703, 72162213705, 72162213709, 68382034610, 68382052106, 68382052110, 50228013910, 62332009310, 71335058202, 68382034616, 68382034705, 68382052116, 65841072110, 65841074905, 65841075005, 65841075030, 43826005703, 43826005709, 42799095401, 42799095402, 72162127603, 72162135503, 72162135509, 71335234601, 71335234602, 71335234603
Memantine	70518329700, 55111059605, 55111059701, 55111059779, 55154415100, 55111059678, 55111059778, 55111059601, 55154763700, 71335160301, 71335160302, 47335003383, 47335032108, 47335032208, 47335032283, 62332007530, 63629195701, 47335032218, 68382054616, 68382054816, 68382054977, 47335003283, 47335003381, 46708045142, 46708045271, 00615826439, 62332007542, 46708045230, 70771111900, 68382054777, 71335160303, 71335160304, 70771132404, 71335034602, 59651040490, 59651040690, 59651040828, 62332007510, 46708045120, 46708045231, 27241007005, 65162078250, 46708045160, 46708045260, 62332007520, 71335189901, 71335189903, 71335189904, 70771111906, 65862065299, 65862065303, 70771112004, 70518333800, 71335186301, 71335186302, 71335186303, 72578000301, 72578000310, 72578000377, 72578000401, 72578000410, 72578000477, 47335003181, 47335003281, 53746017330, 65862065278, 65862065378, 65862065203, 47335003118, 47335003318,

Generic Name	Medication National Drug Code
	71335201301, 71335201302, 70771112001, 70771132203, 53746017310,
	64380077430, 55154266600, 55154266700, 63629195601, 46708045191,
	46708045242, 00527122105, 00527122110, 00527122210, 31722080702,
	53746016910, 70771132103, 70771132209, 70771132409, 71335186304,
	64380077401, 70771132303, 70771132403, 63629739702, 63629739703,
	63629739704, 70771112000, 70771132104, 70771111901, 70771112005,
	70771112006, 64380077403, 70771111904, 70771111905, 71335034601,
	70771132304, 68788773003, 68788773006, 68788773009, 71335179801,
	71335179802, 71335173201, 47335032188, 47335032288, 62332007620,
	65162078350, 65162078450, 70771132109, 70771132309, 71034000730,
	71034000830, 71034000930, 31722080732, 31722080802, 31722080832,
	71335190803, 71335190804, 47335003218, 47335003481, 64380074503,
	64380074530, 68382054677, 33342029712, 33342029715, 33342029812,
	47335003418, 52605007110, 52605007113, 52605007116, 52605007210,
	62332007610, 52605007213, 52605007216, 65162078409, 65162078550,
	46708045110, 46708045130, 46708045210, 46708045220, 65162078209,
	62332007630, 46708045131, 68382054877, 47335003183, 70771132204,
	47335003483, 47335032118, 72189053860, 50268058713, 50268058813,
	72162200305, 72162200306, 72162200405, 72162200406, 69680016330,
	69680016130, 69680016230, 69680016430, 55111059679, 55111059730,
	55111059630, 00615831905, 00615831939, 00615819239, 54569588500,
	54868516100, 54868516101, 54868565400, 62135089560, 62135089660,
	62135094337, 62332007531, 62332007560, 62332007591, 62332007631,
	62332007642, 62332007660, 62332007671, 63629198201, 63629198301,
	63629198401, 63629198501, 63629214201, 63629214301, 63629222201,
	63629222301, 63629251101, 63629251201, 63629251301, 63629251401,
	63629338301, 63629338302, 63629338303, 63629338304, 63629739701,
	63629928601, 27241007006,27241007105, 27241007106, 29300017105,
	29300017116, 29300017205, 29300017216, 31722080760, 31722080860,
	33342006628, 33342029709, 33342029809, 33342029815, 35356010560,
	00904650506, 00904650561, 00904650606, 00904650661, 00904673461,
	00904673561, 00904673661, 00904673761, 64679012102, 64679012103,
	64679012202, 64679012203, 65162016906, 65162017306, 65162078203,
	65162078303, 65162078309, 65162078403, 65162078503, 65162078509,
	65862065260, 65862065360, 65862065399, 66105065003, 66105065103,
	43975024203, 43975024303, 43975026403, 43975026409, 43975026603,
	43975026609, 47335032186, 47335032213, 47335032286, 49848000360,
	49848000460, 49999080430, 49999080460, 50090443400, 50090443401,
	50090443402, 50090583200, 50090583201, 50090583202, 50090592600,
	50090592601, 50090629100, 50090629101, 50090640000, 50090640001,
	50090640002, 10370034611, 10370034709, 10370034711, 10370034811,
	10370034909, 10370034911, 12280028460, 12280038160, 39328055112,
	42291055160, 42291055260, 42292000501, 42292000506, 42292000601,
	42292000606, 43353016518, 43353016553, 43353017018, 43353089718,
	53746016930, 53746016960, 53746017360, 58864088730, 59651040430,
	59651040530, 59651040590, 59651040630, 59651040730, 59651040790,
	60505616205, 60505620803, 60505620903, 60505620909, 60505621003,
	60505621103, 60505621109, 60687017311, 60687017357, 60687018411,
	60687018457, 00121085005, 00121085040, 00456320014, 00456320212,
	00456320511, 00456320560, 00456320563, 00456321011, 00456321060,
	00456321063, 00456340029, 00456340733, 00456341411, 00456341433,
	00456341463, 00456341490, 00456342133, 00456342811, 00456342833,
	00456342863, 00456342890, 00527122106, 00527122205, 00527122206,
	00527194313, 00591387044, 00591387045, 00591387060, 00591387544,
	00591387545, 00591387560, 00591390087, 00832111260, 00832111360,
	68180022907, 68180023007, 68180024606, 68180024706, 68180024709,
	68180024806, 68180024902, 68180024906, 68180024909, 68382054606,
	68382054706, 68382054716, 68382054806, 68382054906, 68382054916,
	70436005404, 70436005504, 70436005506, 70436005604, 70436005704,

Generic Name	Medication National Drug Code
	70436005706, 00378110391, 00378110491, 00378543593, 00378543677, 00378543693, 00378543793, 00378543877, 00378543893, 51407005430, 51407005530, 51407005590, 51407005630, 51407005730, 51407005790, 51407028460, 51407028560, 71335173202, 71335189902, 71335190801, 71335190802, 71610001118, 72189029430, 72189029490, 72578000305, 72578000314, 72578000405, 72578000414, 72603011801, 72603011901, 72603011902, 72606051402, 72606051404, 72606051502, 72606051504, 75839042501, 55111059660, 55111059705, 55111059760, 55289093730, 55289093760, 13668022260, 13668022360, 13668057309, 13925054012, 16590076915, 16590076930, 16590076960, 16714095901, 16714096001, 16714096002, 16714096101, 16714096201, 16714096202, 21695016930, 21695016960, 21695023215, 21695023260
Rivastigmine	51956000107, 51956000207, 51956000307, 62756014761, 71209001403, 71209001303, 71209001310, 55111035201, 46708006471, 46708006671, 65862065005, 55111035501, 33342008906, 33342008912, 33342009006, 33342009012, 33342009106, 33342009112, 33342009206, 33342009212, 62756014561, 55111035230, 46708006310, 46708006410, 46708006491, 46708006630, 65862065078, 65862064978, 46708006360, 46708006391, 55111035401, 65862064905, 65862064878, 65862065105, 65862065178, 46708006510, 62332006310, 62332006330, 62332006371, 62332006391, 62332006410, 62332006430, 62332006471, 62332006491, 62332006510, 62332006530, 62332006571, 62332006591, 62332006610, 62332006630, 62332006671, 62332006691, 55111035330, 55111035430, 55111035530, 71209001503, 46708006560, 46708006660, 72241001110, 71209001510, 55111035301, 62756014661, 62756014861, 71209001210, 46708006371, 46708006430, 46708006591, 46708006691, 65862064805, 46708006610, 46708006330, 46708006460, 46708006530, 46708006571, 71209001203, 71209001410, 72241001210, 72241001310, 72241001410, 72855010001, 72855010101, 72855010201, 54868451200, 54868451201, 54868524000, 54868533900, 54868583900, 54868595400, 54868607000, 54868614500, 62135090060, 62135090160, 62135090260, 62135090360, 62332006360, 62332006460, 62332006560, 62332006660, 62756014513, 62756014586, 62756014613, 62756014686, 62756014713, 62756014786, 62756014813, 62756014886, 63629206401, 63629206501, 63629206601, 63629880701, 63629884601, 33342008909, 33342008915, 33342009009, 33342009015, 33342009109, 33342009115, 33342009209, 33342009215, 35356039430, 12280038960, 00904658761, 00904710761, 47781030403, 47781030411, 47781030503, 47781030511, 47781040503, 47781040511, 63739057610, 63739057710, 63739057810, 63739057910, 65162074934, 65162082534, 65162082634, 65862064860, 65862064960, 65862065060, 65862065160, 60429039360, 60429039460, 60429039560, 60429039660, 60505322006, 60505322106, 60505322206, 60505322306, 60687057401, 60687057411, 00078032306, 00078032315, 00078032344, 00078032361, 00078032406, 00078032415, 00078032444, 00078032461, 00078032506, 00078032515, 00078032544, 00078032561, 00078032606, 00078032615, 00078032644, 00078032661, 00078033931, 00078050115, 00078050161, 00078050215, 00078050261, 00078050315, 00078050361, 00591320860, 00591320960, 00591321060, 00591321160, 00781261406, 00781261413, 00781261460, 00781261506, 00781261513, 00781261560, 00781261606, 00781261613, 00781261660, 00781261706, 00781261713, 00781261760, 00781730431, 00781730458, 00781730931, 00781730958, 00781731331, 00781731358, 68084055001, 68084055011, 70710119601, 70710119607, 70710119701, 70710119707, 70710119801, 70710119807, 00378907016, 00378907093, 00378907116, 00378907193, 00378907216, 00378907293, 51991079306, 51991079406, 51991079506, 51991079606, 51991089730, 51991089799, 51991089830, 51991089899, 51991089930, 51991089999, 72162160803, 72241001103, 72241001203, 72241001303, 72241001403, 75834013305, 75834013360, 75834013405, 75834013460, 75834013505, 75834013560, 75834013605, 75834013660, 55111035205, 55111035260, 55111035305,

Generic Name	Medication National Drug Code
	55111035360, 55111035405, 55111035460, 55111035505, 55111035560, 16714011501, 16714011502, 16714011601, 16714011602, 16714011701, 16714011702, 21695035730, 60687077701
Galantamine	68382017910, 68382017701, 68382017777, 68382017901, 65841075501, 65841075514, 65862045899, 65862045971, 65862074599, 68382017877, 68382017810, 65862074405, 65862074501, 65862074505, 65862074590, 65862045999, 65841075610, 65862074601, 65862074690, 65862074699, 65841075714, 65862045819, 65862046049, 65862046099, 65862074605, 65862074401, 65862074499, 65841075577, 65841075677, 65841075777, 65841075701, 65841075510, 65841075601, 68382017977, 68382017710, 65841075614, 65841075710, 65862074490, 68382017801, 47335083508, 47335083688, 47335083608, 47335083618, 47335083718, 47335083708, 47335083518, 47335083588, 47335083788, 54868503200, 54868511100, 54868545300, 63629236301, 63629236401, 63629236501, 50458038730, 50458038830, 50458038930, 50458039060, 50458039160, 50458039260, 50458039660, 50458039760, 50458039860, 50458039910, 50458049010, 51079046901, 51079046903, 51079047001, 51079047003, 51079047101, 51079047103, 10147088106, 10147088206, 10147088306, 10147089103, 10147089203, 10147089303, 12280029160, 00054009021, 00054009121, 00054009221, 00054013749, 00904710404, 43353090505, 43353098405, 47335083583, 47335083683, 47335083783, 63739070833, 63739099933, 65862045860, 65862045960, 65862046060, 65862074430, 65862074530, 65862074630, 59762000801, 59762000901, 59762001001, 60505254206, 60505254306, 60505254406, 00115112008, 00115112108, 00115112208, 00555013809, 00555013909, 00555014009, 00555102001, 00555102101, 00555102201, 00591349630, 00591349730, 00591349830, 68084049211, 68084049221, 68084072911, 68084072921, 68382017714, 68382017814, 68382017914, 70436000406, 70436000506, 70436000606, 00378272191, 00378272291, 00378272391, 00378810491, 00378810593, 00378810693, 00378810793, 00378810891, 00378811291, 51079085201, 51079085203, 51079085301, 51079085303, 51079085401, 51079085403, 72162144603, 72162144703, 72162144803, 55111040760, 55111040860, 55111040960, 57237004960, 57237005060, 57237005160, 21695018430, 21695059130, 21695078730, 24979072204, 24979072304, 24979072404
Memantine+Donepezil	00456120730, 00456121430, 00456122130, 00456122830, 00456122929, 69238124709, 69238124703, 69238124803<br>69238124809, 69238155203, 69238155209, 69238155303, 69238155309, 00456121411, 00456122811, 00456122904

Supplementary Table 19. Covariate ICD-10 codes.

Outcome	ICD-10 Code
Alcohol abuse	F10.1*, F10.20, F10.21, F10.22*, F10.23*, F10.24, F10.25*, F10.28*, F10.29, Z71.41
Brain tumor	Z85.841, C71.*, C79.3*, D43.0, D43.1, D43.2, D49.6
Chronic diseases[1,10]	
Kidney disease	Based on Charlson definition for renal disease
Heart disease	Based on Charlson definition for myocardial infarction and congestive heart failure
Liver disease	Based on Charlson definition for liver disease (mild, moderate, and severe)
Lung disease	Based on Charlson definition for chronic pulmonary disease
Diabetes mellitus	Based on Charlson definition for diabetes with and without complications
Charlson comorbidity[1,10]	
Myocardial infarction	I21.*, I22.*, I25.2
Congestive heart failure	I09.9, I11.0, I13.0, I13.2, I25.5, I42.0, I42.5-I42.9, I43.*, I50.*, P29.0
Peripheral vascular disease	I70.*, I71.*, I73.1, I73.8*, I73.9, I77.1, I79.0, I79.2, K55.1, K55.8, K55.9, Z95.8*, Z95.9
Cerebrovascular disease	G45.*, G46.*, H34.0*, I60.*-I69.*
Connective tissue disease	M05.*, M06.*, M31.5, M32.*-M34.*, M35.1, M35.3, M36.0
Chronic pulmonary disease	I27.8*, I27.9, J40.*-J47.*, J60.*-J67.*, J68.4, J70.1, J70.3
Peptic ulcer disease	K25.*-K28.*
Diabetes mellitus	E10.*-E14.*
Paraplegia and hemiplegia	G04.1, G11.4, G80.1, G80.2, G81.*, G82.*, G83.0, G83.1*-G83.3*, G83.4, G83.9
Renal disease	I12.0, I12.9, I13.1*, N03.2-N03.7, N05.2-N05.7, N18.*, N19.*, N25.0, Z49.0*-Z49.2*, Z94.0, Z99.2
Liver disease	B18.*, I85.0*, I86.4, I98.2, K70.0, K70.1*-K70.4*, K70.9, K71.1*, K71.3, K71.4, K71.5*, K71.7, K72.1*, K72.9*, K73.*, K74.*, K76.0, K76.2-K76.7, K76.8*, K76.9, Z94.4
Cancer: any malignancy, including lymphoma and leukemia, except malignant neoplasm of skin	C00.*-C26.*, C30.*-C34.*, C37.*-C41.*, C43.*, C45.*-C58.*, C60.*-C76.*, C81.*-C85.*, C88.*, C90.*-C97.*
Metastatic solid tumor	C77.*-C80.*
Dyslipidemia	E78.00, E78.01, E78.1, E78.2, E78.3, E78.4*, E78.5, E78.6, E78.8*, E78.9
Hearing loss[11]	H91.1*, H83.3*, H91.2*, H90*, H91.0*, H91.3, H91.2*, H91.8*, H91.9*
Herpes zoster	B02*, ICD-9 053*
Hypertension	H35.03*, I10, I11*, I12*, I13*, I15*, I67.4, N26.2
Immunocompromising conditions	
Leukemia/lymphoma	C81.*- C86.*, C88.*, C90.*- C96.*, D45, D46.*
Congenital and other immunodeficiencies	D61.09, D61.3, D61.82, D61.9, D70.0, D71, D80.0, D80.1, D80.5, D80.8, D81.*, D82.*, D83.*, D84.0, D84.1, D84.89, D84.9, D89.81*, D89.82, D89.9, E31.0, E70.330, G11.3, Q82.4, Q89.0*
Asplenia/hyposplenia	D57.00, D57.01, D57.02, D57.1, D57.2, D57.20, D57.21, D57.211, D57.212, D57.219, D57.4, D57.40, D57.41, D57.411, D57.412, D57.419, D57.8, D57.80, D57.81, D57.811, D57.812, D57.819, D73.0, Q89.01, Q89.09, Z90.81
HIV/AIDS	HIV Registry
Hematopoietic stem cell/solid organ transplant	Organ transplant or bone marrow transplant record
Mental health disorders	

Outcome	ICD-10 Code
Anxiety	F06.4, F10.180, F10.280, F10.980, F12.180, F12.280, F12.980, F13.180, F13.280, F13.980, F14.180, F14.280, F14.980, F15.180, F15.280, F15.980, F16.180, F16.280, F16.980, F18.180, F18.280, F18.980, F19.180, F19.280, F19.980, F40*, F41*
Depression	F06.31, F06.32, F32.0, F32.1, F32.2, F32.3, F32.4, F32.5, F32.89, F32.9, F33*, F34.1
Metabolic syndrome	E88.81, E88.810
Multiple sclerosis [12]	G35
Parkinson's disease [13,14]	G20*
Huntington's disease	G10
Sleep disorders	F19.182, F19.282, F19.982, F51*, G25.81, G47*, R06.3
Traumatic brain injury [15]	S02.0*, S02.1*, S02.9*, S06.0*, S06.1*, S06.2*, S06.3*, S06.4*, S06.5*, S06.6*, S06.8*, S06.9*
Viral infection [16-19]	
Influenza	J09*-J11*
SARS-CoV-2/COVID-19	U07.1
Other viral infections	B33*, B34*
Other viral pneumonia	J12*, B01.2, B06.81, B25.0
Viral encephalitis	A85*, A86
Viral intestinal infection	A08*
Viral meningitis	A87*, B00.3, B01.0, B05.1, B06.02, B26.1, B27.92
Viral warts	B07*, A63.0

*All subcodes found below the one listed were included in the definition.

AIDS, acquired immunodeficiency syndrome; ICD, International Classification of Diseases; HIV, human immunodeficiency virus

Supplementary References

1. Quan, H., *et al.* Updating and validating the Charlson comorbidity index and score for risk adjustment in hospital discharge abstracts using data from 6 countries. *Am J Epidemiol* **173**, 676-682 (2011).
2. Kim, D.H., Schneeweiss, S., Glynn, R.J., Lipsitz, L.A., Rockwood, K. & Avorn, J. Measuring Frailty in Medicare Data: Development and Validation of a Claims-Based Frailty Index. *J Gerontol A Biol Sci Med Sci* **73**, 980-987 (2018).
3. Salmon, D.A., Black, S., Didierlaurent, A.M. & Moulton, L.H. Commentary on "Common Vaccines and the Risk of Dementia: A Population-Based Cohort Study": Science Can be Messy but Eventually Leads to Truths. *J Infect Dis* **227**, 1224-1226 (2023).
4. Taquet, M., Dercon, Q., Todd, J.A. & Harrison, P.J. The recombinant shingles vaccine is associated with lower risk of dementia. *Nat Med* **30**, 2777-2781 (2024).
5. Xu, S., Ross, C., Raebel, M.A., Shetterly, S., Blanchette, C. & Smith, D. Use of stabilized inverse propensity scores as weights to directly estimate relative risk and its confidence intervals. *Value Health* **13**, 273-277 (2010).
6. Prentice, R.L., Kalbfleisch, J.D., Peterson, A.V., Jr., Flournoy, N., Farewell, V.T. & Breslow, N.E. The analysis of failure times in the presence of competing risks. *Biometrics* **34**, 541-554 (1978).
7. So, Y.L., G.; Johnston, G. Using the PHREG procedure to analyze competing-risks data. in *SAS Global Forum* (Washington, DC., USA, 2014).
8. Nguyen, T.L., *et al.* Double-adjustment in propensity score matching analysis: choosing a threshold for considering residual imbalance. *BMC Med Res Methodol* **17**, 78 (2017).
9. Harding, B.N., *et al.* Methods to identify dementia in the electronic health record: Comparing cognitive test scores with dementia algorithms. *Healthc (Amst)* **8**, 100430 (2020).
10. Quan, H., *et al.* Coding algorithms for defining comorbidities in ICD-9-CM and ICD-10 administrative data. *Med Care* **43**, 1130-1139 (2005).
11. Tonelli, M., *et al.* Associations between hearing loss and clinical outcomes: population-based cohort study. *EClinicalMedicine* **61**, 102068 (2023).
12. Cho, E.B., *et al.* The risk of dementia in multiple sclerosis and neuromyelitis optica spectrum disorder. *Front Neurosci* **17**, 1214652 (2023).
13. Unda, S.R., Antoniazzi, A.M., Altschul, D.J. & Marongiu, R. Peripheral Leukocytosis Predicts Cognitive Decline but Not Behavioral Disturbances: A Nationwide Study of Alzheimer's and Parkinson's Disease Patients. *Dement Geriatr Cogn Disord* **50**, 143-152 (2021).
14. Garcia-Ptacek, S. & Kramberger, M.G. Parkinson Disease and Dementia. *J Geriatr Psychiatry Neurol* **29**, 261-270 (2016).
15. Fann, J.R., *et al.* Long-term risk of dementia among people with traumatic brain injury in Denmark: a population-based observational cohort study. *Lancet Psychiatry* **5**, 424-431 (2018).
16. Levine, K.S., *et al.* Virus exposure and neurodegenerative disease risk across national biobanks. *Neuron* **111**, 1086-1093 e1082 (2023).
17. Lin, H.C., Xirasagar, S., Lee, H.C., Huang, C.C. & Chen, C.H. Association of Alzhemier's disease with hepatitis C among patients with bipolar disorder. *PLoS One* **12**, e0179312 (2017).
18. Huang, S.Y., *et al.* Herpesvirus infections and Alzheimer's disease: a Mendelian randomization study. *Alzheimers Res Ther* **13**, 158 (2021).
19. Lin, C.H., *et al.* Increased risk of dementia in patients with genital warts: A nationwide cohort study in Taiwan. *J Dermatol* **47**, 503-511 (2020).
