# Supplementary material for: Recombinant zoster vaccine is associated with a reduced risk of dementia
Source: Nat Commun. 2026 Feb 9;17:2056. doi: 10.1038/s41467-026-69289-0 (PMC12949228; doi:10.1038/s41467-026-69289-0)
Supplement: Supplementary file 3 — Reporting Summary [file 41467_2026_69289_MOESM3_ESM.pdf]

nature portfolio

Corresponding author(s): Emily Rayens

Last updated by author(s): Oct 6, 2025

Reporting Summary

Nature Portfolio wishes to improve the reproducibility of the work that we publish. This form provides structure for consistency and transparency in reporting. For further information on Nature Portfolio policies, see our Editorial Policies and the Editorial Policy Checklist.

Statistics

For all statistical analyses, confirm that the following items are present in the figure legend, table legend, main text, or Methods section.

n/a	Confirmed	
☐	☒	The exact sample size (*n*) for each experimental group/condition, given as a discrete number and unit of measurement
☒	☐	A statement on whether measurements were taken from distinct samples or whether the same sample was measured repeatedly
☐	☒	The statistical test(s) used AND whether they are one- or two-sided<br>*Only common tests should be described solely by name; describe more complex techniques in the Methods section.*
☐	☒	A description of all covariates tested
☐	☒	A description of any assumptions or corrections, such as tests of normality and adjustment for multiple comparisons
☐	☒	A full description of the statistical parameters including central tendency (e.g. means) or other basic estimates (e.g. regression coefficient) AND variation (e.g. standard deviation) or associated estimates of uncertainty (e.g. confidence intervals)
☐	☒	For null hypothesis testing, the test statistic (e.g. *F*, *t*, *r*) with confidence intervals, effect sizes, degrees of freedom and *P* value noted<br>*Give P values as exact values whenever suitable.*
☒	☐	For Bayesian analysis, information on the choice of priors and Markov chain Monte Carlo settings
☒	☐	For hierarchical and complex designs, identification of the appropriate level for tests and full reporting of outcomes
☒	☐	Estimates of effect sizes (e.g. Cohen's *d*, Pearson's *r*), indicating how they were calculated

Our web collection on statistics for biologists contains articles on many of the points above.

Software and code

Policy information about availability of computer code

Data collection	Epidemiological analyses were conducted using standard commands in SAS 9.4 (SAS Institute; Cary, North Carolina, USA).
Data analysis	Epidemiological analyses were conducted using standard commands in SAS 9.4 (SAS Institute; Cary, North Carolina, USA).

For manuscripts utilizing custom algorithms or software that are central to the research but not yet described in published literature, software must be made available to editors and reviewers. We strongly encourage code deposition in a community repository (e.g. GitHub). See the Nature Portfolio guidelines for submitting code & software for further information.

Data

Policy information about availability of data

All manuscripts must include a data availability statement. This statement should provide the following information, where applicable:

- Accession codes, unique identifiers, or web links for publicly available datasets
- A description of any restrictions on data availability
- For clinical datasets or third party data, please ensure that the statement adheres to our policy

Individual-level data are not publicly available due to privacy concerns and protection of patient identities. Requests for aggregate-level data may be submitted to KPSC and are subject to review. De-identified aggregate-level data that support the findings of this study may be shared upon approval of a proposal and a signed data access agreement.

Research involving human participants, their data, or biological material

Policy information about studies with human participants or human data. See also policy information about sex, gender (identity/presentation), and sexual orientation and race, ethnicity and racism.

Reporting on sex and gender	Sex was considered an important covariate during this analysis. This information was documented by healthcare providers in electronic health records (EHR) during clinical encounters from available medical history on the patient's sex and, if needed, patient self-report<br><br>In our cohort construction, RZV-vaccinated individuals were matched with unvaccinated individuals on sex, as well as age, race/ethnicity, previous vaccination with Zoster Vaccine Live (ZVL), and history of mild cognitive impairment diagnosis. During analysis, we further evaluated the relationship between two doses of RZV and risk of dementia stratified by sex, and found that while reduction in dementia risk was observed in both males and females, the reduction in risk was significantly greater in females.
Reporting on race, ethnicity, or other socially relevant groupings	Race/ethnicity was considered an important covariate during this analysis. This information was documented by healthcare providers in EHR during clinical encounters from patient self-report. Race/ethnicity categories are structured in EHR so providers selected the best fit from a preset list of options.<br><br>In our cohort construction, RZV-vaccinated individuals were matched with unvaccinated individuals on race/ethnicity, as well as age, sex, previous vaccination with Zoster Vaccine Live (ZVL), and history of mild cognitive impairment diagnosis. During analysis, we further evaluated the relationship between two doses of RZV and risk of dementia stratified by race/ethnicity and did not observe any differences in reduced risk of dementia based on racial or ethnic group.
Population characteristics	The mean age was 73.42 (standard deviation [SD]: 6.19) years and 73.33 (SD: 6.69) years in the RZV-vaccinated and unvaccinated populations, respectively. The majority of RZV-vaccinated and unvaccinated individuals were female (57.5% vs 57.7%, respectively) and non-Hispanic White (61.1% vs 61.2%, respectively).
Recruitment	This was a retrospective cohort study
Ethics oversight	This study was approved by the Kaiser Permanente Southern California Institutional Review Board.

Note that full information on the approval of the study protocol must also be provided in the manuscript.

Field-specific reporting

Please select the one below that is the best fit for your research. If you are not sure, read the appropriate sections before making your selection.

☐ Life sciences ☒ Behavioural & social sciences ☐ Ecological, evolutionary & environmental sciences

For a reference copy of the document with all sections, see nature.com/documents/nr-reporting-summary-flat.pdf

Behavioural & social sciences study design

All studies must disclose on these points even when the disclosure is negative.

Study description	We conducted a retrospective cohort quantitative study among KPSC members aged 65 years and older. The RZV-vaccinated group included adults who received two doses of RZV 4 weeks to 6 months apart between 01 April 2018 and 31 December 2020. Vaccinated individuals were 1:4 matched by age, sex, race/ethnicity, and history of ZVL vaccination to unvaccinated individuals who had not received RZV as of the index date. Unvaccinated matches who were later vaccinated with RZV contributed person-time up until they received the first dose of RZV and were then censored.
Research sample	Adults aged 65 years and older at the index date with at least 1 year of continuous KPSC membership before the index date (allowing for a 31-day gap in membership) and until at least 6 months afterwards were eligible for inclusion. Individuals were excluded if they were diagnosed with dementia (by ICD-10 [International Classification of Diseases, Tenth Revision] code; Supplementary Table 16) or were prescribed any medication to treat dementia symptoms prior to or on the index date or within 6 months following the index date (Supplementary Table 17). Additionally, individuals were excluded if they received RZV or ZVL within 6 months following the index date, received the second dose of RZV less than 4 weeks after the first dose, or died within 6 months following the index date. The median age was 72 years old with approximately 58% of the sample identified as female and 61% as non-Hispanic White.
Sampling strategy	Vaccinated individuals were 1:4 matched by age, sex, race/ethnicity, and history of ZVL vaccination to unvaccinated individuals who had not received RZV as of the index date.
Data collection	The index date was defined as the date of the receipt of the second dose of RZV for vaccinated individuals and unvaccinated individuals were assigned the same index date as their vaccinated matches. Follow-up time began 6 months after the index date and lasted until receipt of an additional dose of HZ vaccine, termination of KPSC membership (allowing for a 31-day gap in membership), occurrence of an event of interest, death, or the end of the follow-up period (31 December 2023), whichever occurred first.
Timing	The RZV-vaccinated group included adults who received two doses of RZV 4 weeks to 6 months apart between 01 April 2018 and 31 December 2020, and all individuals (including unvaccinated) were followed until the end of the follow-up period (31 December 2023).

Data exclusions	No data was excluded from analyses
Non-participation	Not applicable; this was a retrospective analysis
Randomization	Not applicable; this was a retrospective cohort study.

Reporting for specific materials, systems and methods

We require information from authors about some types of materials, experimental systems and methods used in many studies. Here, indicate whether each material, system or method listed is relevant to your study. If you are not sure if a list item applies to your research, read the appropriate section before selecting a response.

Materials & experimental systems

n/a	Involved in the study
☒	☐ Antibodies
☒	☐ Eukaryotic cell lines
☒	☐ Palaeontology and archaeology
☒	☐ Animals and other organisms
☒	☐ Clinical data
☒	☐ Dual use research of concern
☒	☐ Plants

Methods

n/a	Involved in the study
☒	☐ ChIP-seq
☒	☐ Flow cytometry
☒	☐ MRI-based neuroimaging

Plants

Seed stocks	*Report on the source of all seed stocks or other plant material used. If applicable, state the seed stock centre and catalogue number. If plant specimens were collected from the field, describe the collection location, date and sampling procedures.*
Novel plant genotypes	*Describe the methods by which all novel plant genotypes were produced. This includes those generated by transgenic approaches, gene editing, chemical/radiation-based mutagenesis and hybridization. For transgenic lines, describe the transformation method, the number of independent lines analyzed and the generation upon which experiments were performed. For gene-edited lines, describe the editor used, the endogenous sequence targeted for editing, the targeting guide RNA sequence (if applicable) and how the editor was applied.*
Authentication	*Describe any authentication procedures for each seed stock used or novel genotype generated. Describe any experiments used to assess the effect of a mutation and, where applicable, how potential secondary effects (e.g. second site T-DNA insertions, mosiacism, off-target gene editing) were examined.*
